# LSTM based stock prediction using weighted and categorized financial news

**DOI:** 10.1371/journal.pone.0282234

**Published:** 2023-03-07

**Authors:** Shazia Usmani, Jawwad A. Shamsi

**Affiliations:** Systems Research Laboratory, FAST-National University of Computer and Emerging Sciences, Karachi, Pakistan; Indian Institute of Technology Patna, INDIA

## Abstract

A significant correlation between financial news with stock market trends has been explored extensively. However, very little research has been conducted for stock prediction models that utilize news categories, weighted according to their relevance with the target stock. In this paper, we show that prediction accuracy can be enhanced by incorporating weighted news categories simultaneously into the prediction model. We suggest utilizing news categories associated with the structural hierarchy of the stock market: that is, news categories for the market, sector, and stock-related news. In this context, Long Short-Term Memory (LSTM) based Weighted and Categorized News Stock prediction model (WCN-LSTM) is proposed. The model incorporates news categories with their learned weights simultaneously. To enhance the effectiveness, sophisticated features are integrated into WCN-LSTM. These include, hybrid input, lexicon-based sentiment analysis, and deep learning to impose sequential learning. Experiments have been performed for the case of the Pakistan Stock Exchange (PSX) using different sentiment dictionaries and time steps. Accuracy and F1-score are used to evaluate the prediction model. We have analyzed the WCN-LSTM results thoroughly and identified that WCN-LSTM performs better than the baseline model. Moreover, the sentiment lexicon HIV4 along with time steps 3 and 7, optimized the prediction accuracy. We have conducted statistical analysis to quantitatively assess our findings. A qualitative comparison of WCN-LSTM with existing prediction models is also presented to highlight its superiority and novelty over its counterparts.

## 1. Introduction

News analysis could play a significant role in the prediction of stock trends due to the fact that the stock market is heavily influenced by market-related news [1]. News analysis with deep insight could generate significant benefits by improving stock prediction performance. In recent years, stock related news is analysed from different perspectives but there is still much room to mine information from financial news repository. However, the task of news analysis is challenging due to many factors.

First and foremost, proper categorization of financial news is important so that news can be assessed precisely in its area of influence. For instance, Schumaker and Chen partitioned financial news articles into two news groups related to similar industries and sectors and found that sector-based grouping enhanced prediction model’s performance [2]. Inspecting the significance of using multiple and simultaneous news groups in predicting stock trends has been a research-oriented task in the context of news analysis as well. Shynkevich, et al. [3] have shown improvement in prediction accuracy by concurrent and appropriately weighted incorporation of news groups into the prediction model. Hence, news groups should be identified according to their area of influence. Furthermore, there should be a way to identify the optimized weights for the impact of each news group. And finally, an efficient machine learning approach should be investigated that simultaneously incorporates these categorized and weighted information in order to improve prediction performance.

Selecting an efficient way to extract information from news and representing it in machine-readable format is another research-oriented task. Textual analytics deals with text processing to extract significant information [4]. Sentiment analysis is a form of text analytics that measures the polarity of text. It is a way to identify the meaning of the text in terms of how positive or negative it is [5]. A few researchers have also used sentiment analysis for stock prediction [6, 7]. Sentiment dictionaries play a vital role in measuring sentiment scores. These dictionaries can be general or specific for a domain, like Harvard IV (HIV4) and Loughran and McDonald (LM) are used as general and specific domain dictionaries. So there is a need for comparative analysis between different sentiment lexicons to achieve optimized prediction performance.

Most of the earlier work rely only on the input data at time point *t* to predict stock trend at time point *t+1*. Recently, many studies adopted the stock prediction problem as a sequence learning problem where the input to the prediction model is a sequence of input at successive time points [8–10]. But there is little work that investigates the effectiveness of multiple input sequence length in quest of enhancing prediction performance. Moreover, adopting an efficient machine learning model that could efficiently maintain memory across long sequences to improve prediction accuracy is important to implement sequence learning.

Our research objective is motivated by the above mentioned opportunities and challenges in the quest of enhancing the performance of news sensitive stock prediction model.

### 1.1 Research objectives

In order to improve the performance of news sensitive stock trend prediction, we have identified the following objectives of our research:

1. To propose and evaluate an extensive model, which can cater complexities of the hybrid input dataset in order to extract significant information.
2. To incorporate the filtered news groups into the prediction model simultaneously with their learned weight.
3. To effectively identify sentiments of news to predict the stock trend.
4. To adopt sequence learning in stock prediction to utilize historical sequence information.

The above research objectives lead towards the proposal of a Long Short-Term Memory (LSTM) based Weighted and Categorized News (WCN-LSTM) stock prediction model. WCN-LSTM integrates state of the art features. The proposed model utilizes hybrid input. It allows the incorporation of multiple weighted news groups simultaneously into the prediction model. WCN-LSTM performs feature extraction from news using lexicon-based sentiment analysis. It uses the LSTM layer to process sequential input data. The implementation of WCN-LSTM causes to raise the following empirical research questions.

### 1.2 Research questions

**RQ1:** Does news categorization give more insight into understanding news impact on the stock market in turn improving prediction accuracy?

**RQ2:** Do different news categories have different weights of their impact on the stock market to significantly enhance prediction performance?

**RQ3:** How to identify the optimized value of weights for categorized news impact?

**RQ4:** Which sentiment dictionary improves WCN-LSTM performance significantly? general or domain specific?

**RQ5:** What should be the optimized time step value of the input sequence for WCN-LSTM to predict the trend of the next day for a stock?

### 1.3 Contributions

We have proposed a new stock prediction model. Moreover, to demonstrate the significance of the proposed model in improving stock prediction accuracy our contributions are given below:

1. We have selected Pakistan Stock Exchange (PSX) in order to perform experiments. Stock prices are downloaded from the website of PSX Stock for the period of January 2006 to August 2018. News headlines are scraped from a newspaper (*The News*) archive along with the publishing date. News headlines are categorized using an unsupervised classification technique suggested by Usmani and Shamsi [11].
2. We have implemented our proposed stock prediction model, WCN-LSTM. Moreover, to perform a quantitative analysis, we have also implemented a baseline model proposed by Li, et al. [8].
3. We have performed extensive experiments by utilizing different sentiment lexicons and varying input sequence lengths, to reveal significant findings for the case of PSX.
4. We have adopted Wilcoxon signed-rank test to perform statistical analysis for all experimental scenarios (see section 6.2).
5. We have also presented a qualitative analysis between WCN-LSTM and existing stock prediction models to show the significance and novelty of our proposed model (see Fig 13).

**Fig 1:**
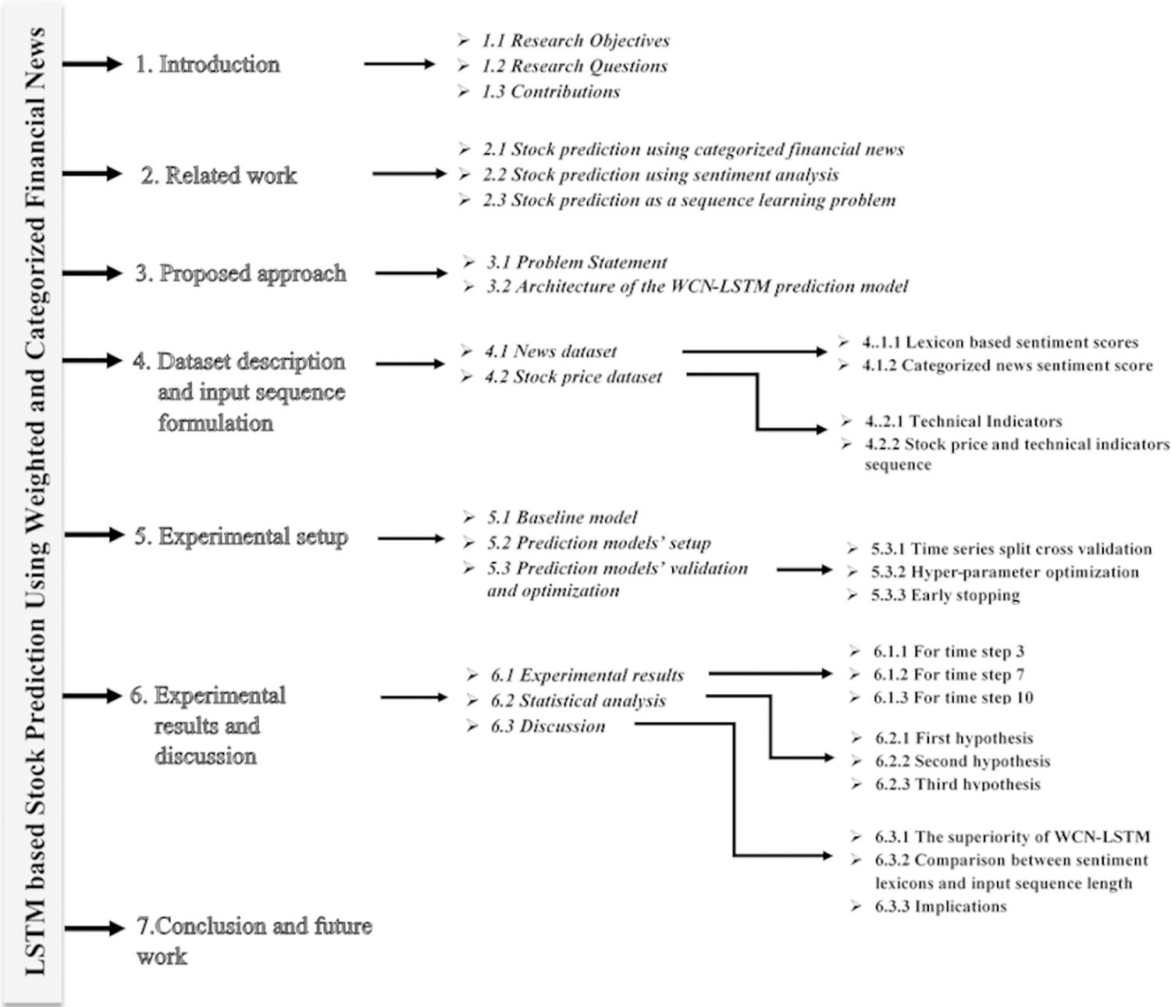
Paper organization.

For evaluation, we have adopted accuracy and F1-score as metrics. We have observed that our proposed approach performs better than the baseline approach by combining the impact of news categories according to their learned weights. The empirical research questions are answered by employing empirical and statistical evidence in the last section of the paper. The paper is organized as follows. Section 2 discusses the literature review. The proposed approach is discussed in section 3. The dataset description along with input preparation is presented in section 4. The experimental setup and results are discussed in sections 5 and 6, respectively. Finally, section 7 concludes this research along with future work. The organization of the paper is shown in Fig 1.

## 2. Related work

News sensitive stock trend prediction is addressed in the literature mostly by using stock price data along with the news. Moreover, technical indicators are also used frequently which are derived from stock price data. In [12], the authors discussed the importance of hybrid information extracted from stock price time series and news to improve prediction performance. A few researchers have shown the significance of incorporating hybrid information into the prediction model in terms of accuracy [13–15]. Moreover, it has also been shown that deep learning architectures improve feature representations [16] and prediction performance [17] in the financial domain. In this section, literature related to stock prediction is discussed briefly from the perspective of news categorization, sentiment analysis, and sequence learning.

### 2.1 Stock prediction using categorized financial news

Mostly stock prediction deals with only one news category which influences the stock market. In literature, only few authors addressed the significance of incorporating categorized news in the prediction model. In [13], authors employed SVM for prediction and incorporated general market and company specific news along with technical indicators. They showed that prediction performance and system profitability were enhanced by taking in multiple news categories and technical indicators. However, they considered the news impact on the market for only 24 hours of its release. Although there could be some news that has long-term impact like news about government policies for the financial market.

There are different industry classification standards to group companies with a similar output to take the advantage of investigating their group effect. Global Industry Classification Standard (GICS) is one of the industry classification standards and employs four level hierarchy of Sector, Industry Group, Industry and Sub-Sector [2, 3].

Schumaker and Chen [2] employed GICS to partition news articles according to their relevance to sector, industry, subindustry, etc. They have used these news groups to identify their effect on stock prediction performance. They have found that prediction performance varies for different news group. However, they have used only one news group at a time to investigate its effect on prediction performance. For instance, the whole news dataset, sector-related news group, industry related news group etc. are incorporated in prediction model and they found that sector-related news group performed better among all news groups. Moreover, they employed Support Vector Regression (SVR) machine learning algorithm for prediction. Furthermore, news groups have not been incorporated simultaneously into the prediction model that is, the combined impact of all news groups has not been investigated in their work.

In [3], authors adapted this approach by incorporating all identified news groups simultaneously in the prediction model. They showed how properly weighted news articles with different degrees of relevance with stock prices used simultaneously can improve prediction performance significantly. They used the Multiple kernel learning (MKL) approaches for stock prediction by integrating the information coming from the prediction using a separate kernel for each news group. They have used newsgroups based on GICS standard which has some lacking in finding group of relevant articles.

In [18], the authors claimed that heterogeneity exists in GISC that limits the relevant finding regarding stock prediction. They proposed a model that searches for a group of companies with high relevance. They showed that the proposed model outperforms GISC system based prediction. However, these newsgroups are not belonging to the structural hierarchy of the stock market and their degree of relevance has not been learned ae well.

### 2.2 Stock prediction using sentiment analysis

Stock market-related news not only states the current market status but also has an impact on market volatility. There is a lot of work exists where market-related textual data is mined to extract sentiments about the financial market. There are two major ways to perform sentiment analysis: classification algorithm based and sentiment lexicon-based.

In the classification algorithm based approach labeled data is used to train the algorithm. Jiawei and Murata [6] used training data with positive and negative sentiment labels. LSTM is adopted for sentiment analysis and trend prediction. Multiple news articles at day *t* form an input sequence and are passed into the sentiment analysis module to generate a sentiment label at day *t*. Technical indicators after dimensionality reduction are passed along with sentiment labels into the trend prediction module. This module predicts the average trend of the next three days from day *t* and achieves 66.32% accuracy. Although they have proved the effectiveness of sentiment analysis by improving prediction performance, they have not utilized the strength of the LSTM model by passing input data of succeeding days. Carosia, et al. [7] performed tweets sentiment analysis using Multi-Layer Perceptron (MLP) for Brazilian stock market. Tweet sentiments are investigated using an absolute number of tweets, weighted tweets sentiments by favorites, and weighted tweets sentiments by retweets. Comparative analysis showed that MLP outperformed other machine learning models. They have considered only tweets sentiments as input data for market movement prediction which raises questions on prediction accuracy. Because there may be tweets about past moves and users may use multiple accounts for the same tweets.

Sentiment analysis can also be approached by using sentiment lexicons. These lexicons are created manually using rules and vocabulary [19, 20]. These manually created lexicons are small in size in turn limited the performance of prediction algorithms. In order to increase the size of sentiment lexicons, semi-automated approaches are proposed in the literature where manually created small-sized lexicons are used as seeds for automated approaches [21]. For instance, SentiWordNet 3.0 [22] and SenticNet 5 [23] are semi-automatically created sentiment lexicons. Sentiment lexicons are general and for a specific domain. For instance, Vader [19] and Harvard IV (HIV4) are general sentiment lexicons while LM is specific for the financial domain.

Li, et al. [24] adopted HIV4 and LM sentiment lexicons to generate news sentiment scores. They also used stock price data along with sentiment scores. Support Vector Machine (SVM) is used for making stock trend predictions. They have shown that sentiment scores enhanced prediction accuracy as compared to the Bag of Words (BoW) feature representation. However, the difference between the prediction accuracy of HIV4 and LM was insignificant. Although, it is mentioned that there are two groups of market-related and stock-related news. But they have not captured their impact separately so that deep insight could be explored.

Picasso, et al. [25] used technical indicators and news sentiment as input. They adopted a feed-forward neural network for stock trend prediction. News sentiment analysis is performed using LM and AffectiveSpace 2. They have examined the effectiveness of different feature sets. However, they have used a small-sized dataset not enough for comparative analysis.

Li, et al. [8] used four different manuals and semi-automatically created sentiment lexicons. They performed stock trend prediction for Hong Kong Stock Exchange. The proposed LSTM based stock prediction model incorporates stock prices, technical indicators, and news sentiment scores. They showed that hybrid input enhanced prediction accuracy. Moreover, sentiment lexicon specific for the financial domain performed better than other sentiment lexicon-based prediction performances. They have used LSTM layers in prediction model and incorporated sequential input data. But experiment to investigate the optimized time step for the input sequence was missing.

### 2.3 Stock prediction as a sequence learning problem

The problem of stock prediction is always challenging for the research community due to its high volatility. However, recent development in deep learning models opens new ways to tackle this type of data from different perspectives. For instance, Convolutional Neural Network (CNN), Recurrent Neural Network (RNN), LSTM, etc. are efficiently used in literature.

Ding, et al. [26] extracted event-based textual features from the news. They improved the quality of extracted events using embedding and knowledge bases. They employed CNN for prediction and showed that their proposed approach for event extraction enhanced prediction accuracy. However, they have not taken advantage of stock price data along with textual features for stock trend prediction.

Vargas, et al. [14] input hybrid information into a prediction model based on hybrid deep learning approaches. Textual features are extracted using word and sentence embedding and technical indicators are derived from stock price data. The prediction model is built by combining CNN and LSTM layers and incorporating the previous day’s information in order to predict the stock trend of the next day. They showed that prediction accuracy is improved when technical indicators are also added to the input set along with the news titles. But they have not combined observations from successive days to form an input sequence that can be efficiently processed by LSTM.

Hu, et al. [9] incorporated hybrid information and utilized a hybrid deep learning model for news-oriented stock trend prediction. They adopted Gated Recurrent Unit (GRU) for sequential modeling. GRU is a variant of RNN. The authors [10], utilized stock price with a news sentiment score. They showed that analyzing the lengthy input sequences can significantly improve the accuracy of the LSTM based model. Li, et al. [8] input hybrid sequential information into the LSTM based prediction model. Pokhrel, et al. [27] performed a comparative study between CNN, LSTM, and GRU to predict closing price for Nepal Stock Exchange. They used Root Mean Square Error (RMSE) as a performance evaluation metric while the input sequence comprises stock prices, macroeconomics data, technical indicators and sentiment scores of financial news. They found that LSTM performed better than CNN and GRU. Li and Pan [28] used ensemble of LSTM and GRU deep learning models to learn from sequential data. They showed that ensemble learning significantly improves prediction performance. However, the above review in the context of the sequence learning problem shows that the optimized length of sequential data is not investigated for the problem under consideration. Moreover, hybrid deep learning models should be adopted to effectively enhance prediction performance.

In this discussion, we have found that previous approaches have recognized the significance of hybrid information for stock prediction. News processing using sentiment analysis based approaches have gained widespread interest in recent years because it allows to make faster and accurate conclusions. In stock prediction, sentiment analysis is mostly adopted by employing sentiment lexicons. However, research is going on for enrichment in sentiment lexicons by utilizing semi-automated and state of the art approaches.

News plays a vital role in stock prediction. Despite that, financial news categorization at a more granular level according to the structural hierarchy in the stock market is not addressed in the literature. Although, categorized news opens new perspectives to investigate news impact more deeply.

By addressing the research gap and considering the state of the art techniques, we propose an approach to predict the trend of the next day for target stock by employing information from previous days. Our proposed approach utilizes hybrid information as model input, employs sentiment analysis as a text mining technique, and incorporates categorized news into the prediction model along with their learned weights. Consecutive input vectors are combined to form a sequence according to a given time step and passed into the model. Moreover, the proposed model adopts LSTM layers to perform sequential learning. The proposed approach is discussed in the next section.

## 3. Proposed approach

We propose an LSTM based Weighted Categorized News (WCN-LSTM) stock trend prediction model. The WCN-LSTM model utilizes input data from textual and numerical sources and performs binary classification. It strengthens the prediction approach by using sequential data along with weighted news sentiment scores for different news categories. The WCN-LSTM is formulated as given below:

### 3.1 Problem statement

Given a stock *s* with close price, volume, technical indicators, market news sentiment score, sector news sentiment score, and stock news sentiment score over a lookback window of *n* days over the day range [*t-n*, *t-1*]. The stock trend for stock s from day *t-1* to day *t* is defined as in Eq 1:

$$\mathrm{Trend}=\left\{ \begin{array}{c} 0,\;{\mathrm{Close}\_\mathrm{Price}}_{t‐1}\geq {\mathrm{Close}\_\mathrm{Price}}_{t}\\ 1,\;{\mathrm{Close}\_\mathrm{Price}}_{t‐1}<{\mathrm{Close}\_\mathrm{Price}}_{t} \end{array}\right.$$ (1)

We formulate the problem as in Eq 2:

$$\mathrm{Dense}:[\left\{\left(\mathrm{LSTM}:\delta_{\mathrm{seq}}\right)\},\{\alpha (\mathrm{LSTM}:{\theta 1}_{\mathrm{seq}}),\beta (\mathrm{LSTM}:{\theta 2}_{\mathrm{seq}}),\gamma (\mathrm{LSTM}:{\theta 3}_{\mathrm{seq}})\right\}]\to \{\mathrm{Trend}s\}$$ (2)

Where

*δ* = Stock close price, volume, and technical indicators

*θ1* = Market-related news sentiment scores

*θ2* = Sector-related news sentiment scores

*θ3* = Stock-related news sentiment scores

*α*, *β*, and *γ* are weights for *θ1*, *θ2*, and *θ3*.

While

α+β+γ=1 (3)

Where

Trend = set of prediction labels

LSTM and Dense are neural network layers, used to predict stock trends.

The impact of financial news is equally important as the impact of stock price data in stock trend prediction. In our scenario, we have categorized financial news into three news groups according to the stock market structural hierarchy. Hence, there is a constraint that the total weight of categorized financial news has to be considered as 1 and the weight of each news category is learned through the training dataset. Eq 3 represents that sum of the total weights is equal to 1.

### 3.2 Architecture of the WCN-LSTM prediction model

The architecture of the WCN-LSTM is comprised of multiple neural network layers. In WCN-LSTM, each sequential input is passed to the LSTM layer. LSTM is a type of neural network deals with sequential data where the next observation is dependent on previous observations in a sequence. It can support short-term as well as long-term dependencies in a sequence by using its gating mechanism [5, 29]. In our scenario, the LSTM cell is adopted according to the baseline approach proposed in [8] where the sigmoid activation function is replaced with the hard sigmoid activation function.

The dropout layer tackles the issue of overfitting in the deep neural network which suffers from this issue due to the small dataset for training. In our model, the dropout layer is added after each LSTM layer so it probabilistically excludes some input vector and recurrent connections to LSTM [8]. The concatenate layer is responsible to concatenate the input vectors coming from different tracks into an output vector.

The dense layer is a neural network layer that is connected deeply, which means each neuron in the dense layer receives input from all neurons of its previous layer. The dense layer is found to be the most commonly used layer in the models. In our WCN-LSTM, it is the last layer and contains a sigmoid activation function which is specifically used for binary classification problems.

**Fig 2:**
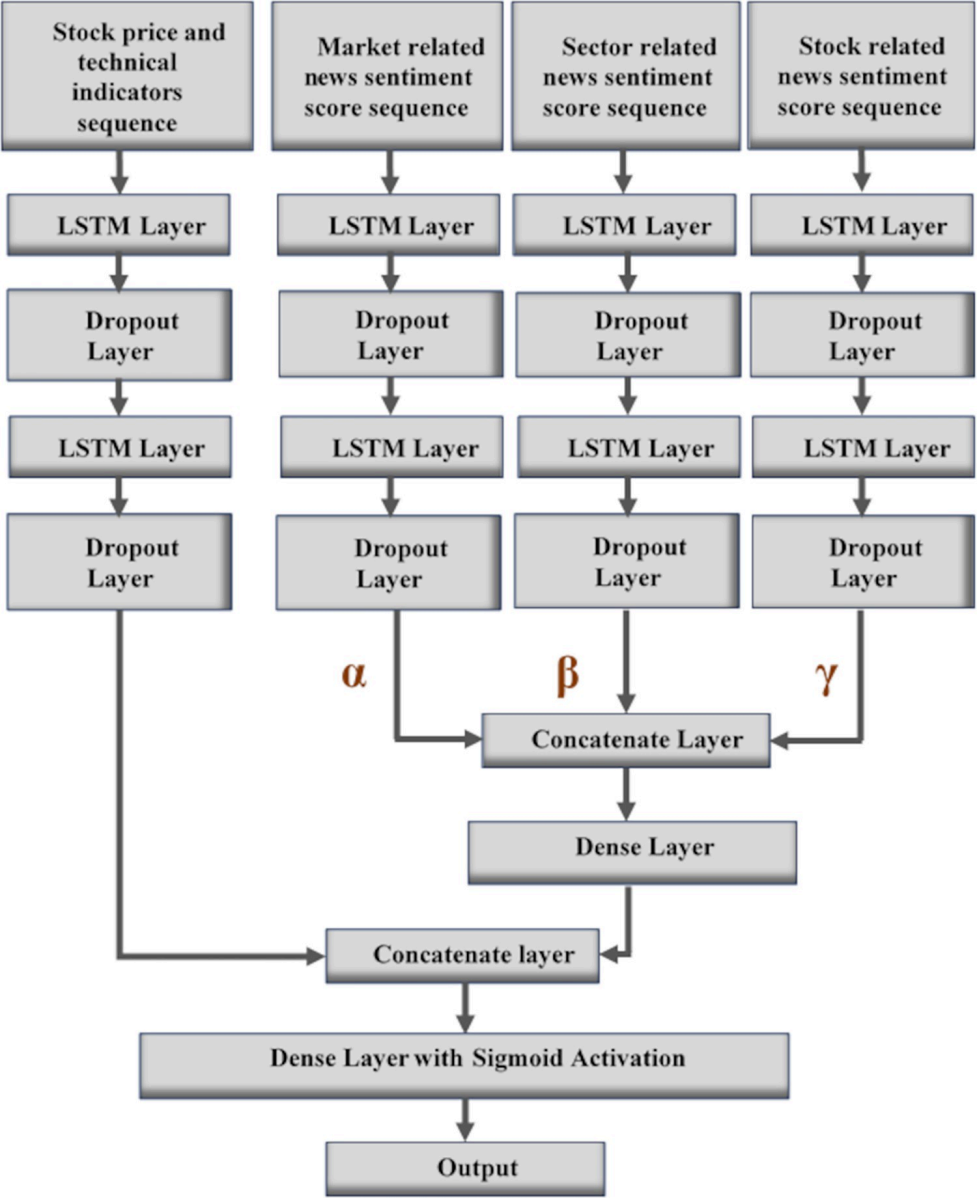
Architecture of the LSTM based Weighted Categorized News (WCN-LSTM) prediction model.

We have combined all layers discussed above and presented our proposed prediction model WCN-LSTM. The architecture of our proposed model is demonstrated in Fig 2.

## 4. Dataset description and input sequence formulation

WCN-LSTM utilizes the news and stock price dataset. This section describes datasets along with the step of feature extraction and formulation of input sequences.

### 4.1 News dataset

There are two ways to perform text categorization, by adopting automatic classification algorithms or manually assigning categories to each news. Classification algorithms required enough amount of labelled data for training [30, 31].

If there is no labelled data, manual effort is required to perform news categorization which is a tiring job. There are approaches that reduce manual efforts by introducing the semi-automatic way of text classification. These approaches manually define some domain related keywords, extend the list of keywords using NLP techniques, and then utilize these keywords in clustering methods [32, 33].

We are considering news headlines as textual input data rather than complete news articles for text categorization. The authors Chen, et al. [34] suggested that news headlines contain less noise and more valuable information than news bodies. In [14, 35, 36], news titles are suggested to perform experiments.

Publically available news headlines are scrapped from 2006 to 2018 and then arranged into three different groups. In order to categorize financial news headlines according to their area of influence, we considered the structural hierarchy of the stock market where the stock market has multiple sectors and each sector has multiple stocks.

In [11], news headlines related to Pakistan Stock Exchange (PSX) are filtered and categorized according to the structural hierarchy of the stock market using a proposed semi-automatic approach. News headlines are divided into three news groups. The first group contains news related to the whole stock market. The second group contains news related to the specific sector and the third group holds news related to a specific stock. News headlines are aligned with their publishing date. For any specific date, there might be news related to all three news groups or maybe only for one or two news groups. In Table 1, all news groups and their descriptions are given. PSX has many sectors but for this research work, a limited number of leading sectors are considered. News headlines related to these sectors are labeled according to the approach proposed in [11] for unlabeled data.

**Table 1:** News groups in the stock market.

News Group	Description
Market News	This group or category contains general news related to the overall stock market that is a news related to PSX.
Sector News	This group represents news categories related to the leading PSX sectors. For instance, Oil & Gas sector, Textile sector, and Refinery sector, etc.
Stock News	This group covers news categories related to active stocks in leading PSX sectors. For instance, PSO, KEL, BYCO, HBL, etc.

PSX or KSE-related news headlines belong to the first news group where news represents whole stock market. While all selected sectors-related news belongs to the second news group. Whereas, all active stocks in selected sectors belong to the third news group. In Table 2, all news categories along with their number of filtered news headlines is illustrated.

**Table 2:** Number of news headlines in news categories.

Market	News headlines	Sector	News headlines	Stock	News headlines
**Pakistan Stock Exchange**	2616	Oil & Gas	1717	APL	8
				BPL	0
				HASCOL	17
				HTL	0
				MARI	26
				OGDC	58
				POL	297
				PPL	149
				PSO	340
				SHEL	139
				SNGP	1
				SSGC	176
		Textile	2692	GATM	16
				NML	23
		Technology & Communication	399	AVN	5
				NETSOL	23
				PAKD	1
				PTC	326
				SYS	2
				TPL	20
				TRG	11
		Power Generation & Distribution	620	EPQL	5
				HUBC	6
				KAPCO	2
				KEL	113
				NPL	4
				PKGP	1
		Refinery	112	ATRL	4
				BYCO	41
				NRL	14
				PRL	16
		Commercial Banks	1155	ABL	39
				AKBL	19
				BAFL	94
				HBL	171
				MCB	176
				NBP	330
				SCBPL	58
				SIILK	44
				SNBL	33
				UBL	173

#### 4.1.1 Lexicon based sentiment scores

We have selected three lexicons to generate a sentiment score vector for news headlines. Vader and HIV4 are general purpose lexicons while LM is specifically used in the financial domain.

#### 4.1.2 Categorized news sentiment scores sequence

The sentiment score vector is generated for each news category. Market, sector, and stock news sentiment score vectors are termed θ1, θ2, and θ3 respectively. Sequences for all three news categories vectors are generated according to given *n* previous days for the day range [*t-n*, *t-1*] as:

$$\theta 1_{seq}‐>\{\theta 1_{t‐n},\;\ldots \ldots .,\;\theta 1_{t‐2},\;\theta 1_{t‐1}\}$$ (4)

$$\theta 2_{seq}‐>\{\theta 2_{t‐n},\;\ldots \ldots .,\;\theta 2_{t‐2},\;\theta 2_{t‐1}\}$$ (5)

$$\theta 3_{seq}‐>\{\theta 3_{t‐n},\;\ldots \ldots .,\;\theta 3_{t‐2},\;\theta 3_{t‐1}\}$$ (6)

Where *n* = lookback days

Eqs 4, 5, and 6 represent sequences of sentiment scores for the market, sector, and stock-related news categories.

### 4.2 Stock price dataset

Stock price data contains different attributes like open price, close price, volume, etc., and is aligned with the publishing date. We have selected close price and volume as input features. Furthermore, the dataset is processed to derive some new attributes. We have to perform binary classification to perform stock trend prediction. So the target variable *trend* is calculated according to the Eq 1.

#### 4.2.1 Technical indcators

Technical indicators are data points derived from historic stock prices and represent future price trends. They are significantly used in literature along with other textual and numerical input data [8, 13, 25]. We have used ten technical indicators suggested by Li, et al. [8]. The adopted indicators are shown in Table 3.

**Table 3:** Technical indicators selected for stock trend prediction (Adopted from [8]).

Technical Indicator	Description
MA10	10-day close price moving average
MA20	20-day close price moving average
MA30	30-day close price moving average
MACD (DIFF)	The difference between EMA12 and EMA26
MACD (DEA)	9-day exponential moving average of DIFF
MACD	Moving average convergence and divergence
RSI6	6-day relative strength index
RSI12	12-day relative strength index
RSI24	24-day relative strength index
MFI	Money flow index

#### 4.2.2 Stock price and technical indicators sequence

Input vector *δ* contains stock close price, volume, and ten different technical attributes. For the detail of technical indicators the work of Li, et al. [8] is suggested.

Furthermore, this module is employed to generate an input sequence according to a given number of time steps. Let’s say to predict the stock trend of day *t*, a sequence is generated from price vector *δ* according to given *n* previous days for the day range [*t-n*, *t-1*] as:

δ={Close_Price,Volume,Technicalindicators} (7)

$$\delta_{seq}‐>\{\delta_{t‐n},\;\ldots \ldots .,\;\delta_{t‐2},\;\delta_{t‐1}\}$$ (8)

Where *n* = lookback days

Eqs 7 and 8 represent the input vectors and sequence containing the stock price and its derived information.

## 5. Experimental setup

In this section, baseline model architecture, setup of both models along with hyper-parameter tuning are presented. Moreover, prediction models are evaluated using performance measures. For binary prediction problems, accuracy, precision, recall, and F1-score are commonly used as evaluation metrics. We have selected accuracy and F1-score performance measures to evaluate the prediction model’s performance.

### 5.1 Baseline model

We have adopted a baseline model from [8] for the binary prediction of stock trends. Financial news is used to calculate sentiment scores without further categorization. Stock’s close price, volume, and technical indicators are used along with news sentiment scores. The input sequence generated from stock price data is the same as formulated in Eqs 4 and 5. The news sentiment score vector is termed as θ and the sequence generated from news sentiment scores is represented as in Eq 9:

$$\theta_{seq}‐>\{\theta_{t‐n},\;\ldots \ldots .,\;\theta_{t‐2},\;\theta_{t‐1}\}$$ (9)

**Fig 3:**
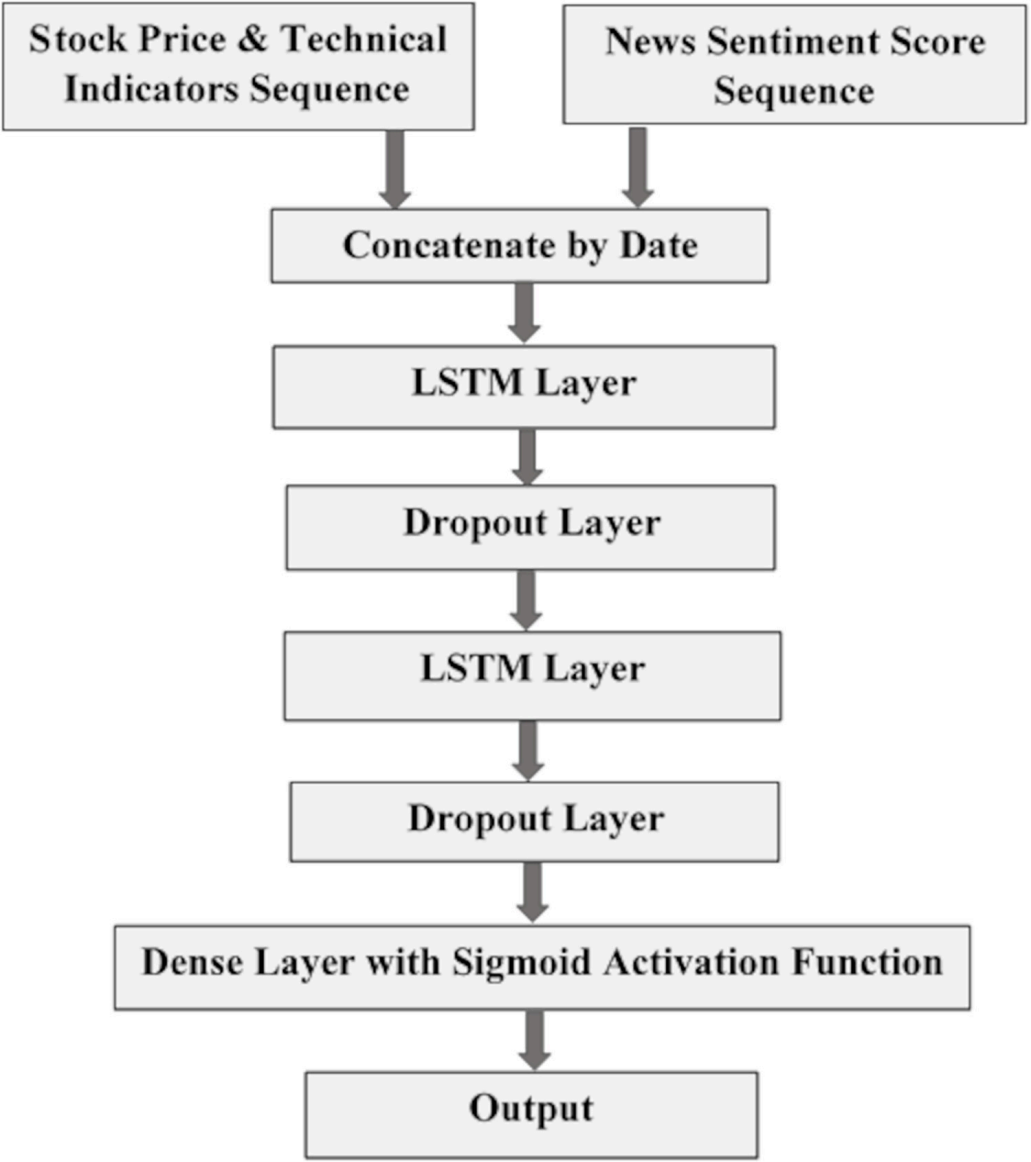
Architecture of the baseline prediction model.

In the baseline model, two sequences of stock price data and news sentiment scores are passed as input into the model. Input sequences are passed into the concatenation layer where both sequences are combined and observations in sequences are aligned according to publishing date. Our WCN-LSTM model is adapted from the baseline model and detail related to the neural network layers have been discussed in section 3. The architecture of the baseline model is demonstrated in Fig **3**.

### 5.2 Prediction models’ setup

In order to evaluate the neural network based prediction model, we have selected binary cross-entropy as a loss function in our binary classification problem. The neural network model’s training is performed by an optimizer algorithm. we have selected Root mean square prop (RMSProp), suggested by [37] for recurrent neural network. Learning rate is a critical hyper parameter of an optimizer algorithm. It defines the step size of each iteration in optimization algorithm while approaching the minima of loss function. Initially learning rate is set to 0.001. Moreover, a learning rate deduction technique is employed to adjust it according to the change of loss. After five consecutive epochs, if no change in loss, the learning rate is reduced to one tenth of itself [8]. Batch size and the maximum number of epochs are set to 32 and 500 for both prediction models.

### 5.3 Prediction models’ validation and optimization

In order to validate and optimize prediction model performance, we have performed model training using cross validation and adopted the grid search technique in search of optimizing the model’s hyper-parameters. We have also incorporated an early stop mechanism to improve generalization and to reduce overfitting of the deep learning model.

To accomplish the model tuning process, we have divided the dataset into three parts. The first part contains 70% of the total data and is used for model learning. While the remaining data is divided into two equal parts used for model testing and implementing an early-stop mechanism.

**5.3.1 Time series split cross validation.** In stock prediction, data related to the stock market is treated as a time series. That is observations are collected at regular intervals of time. Mathematically, it is described as in Eqs 5,6,7,8, and 15 for numerical and textual data. For time series data, the shuffling of data is incorrect to validate the model’s performance. Consequently, time series split cross validation is suggested in the literature by Ratto, et al. [38] for stock prediction. In a time split cross validation scheme, training and validation sets are selected in each iteration so that the validation set is always ahead of the training set. Likewise, we have adopted time series cross validation and divided the training set into 3-folds.

#### 5.3.2 Hyper-parameter optimization

In machine learning, hyper-parameters are the model’s parameters that control the learning model. Hyper-parameter optimization is the process to select the best combination of hyper-parameters values so that performance of the learning model can be optimized.

We have adopted a grid search technique to search for the best value for hyper-parameters. We have performed a grid search for baseline and WCN-LSTM and identified the best values by considering the models’ accuracy from the candidate values. It is shown in Tables 4 and 5 for baseline (LSTM) and WCN-LSTM prediction models.

**Table 4:** Baseline model’s (LSTM) optimized hyper-parameters values.

Hyper-parameters	Description	Candidate Values	HIV4			LM			Vader		
			Time step			Time step			Time step		
			3	7	10	3	7	10	3	7	10
Neurons	Number of neurons in hidden layer	{20,50,100,200}	50	200	50	100	100	200	20	50	20
Dropout rate	Dropout rate for dropout layers	{0.2,0.35,0.5}	0.5	0.35	0.5	0.2	0.2	0.2	0.35	0.5	0.35
Kernel initializer	Method for initializing weight matrix of input features	{RandomNormal, RandomUniform, glorot uniform, glorot normal}	glorot_ uniform	glorot_ uniform	glorot_ uniform	glorot_ uniform	Random Normal	Random Normal	glorot_ uniform	Random Normal	glorot_ uniform
Kernel regularizer	Regular function applied to the weight matrix of kernel	{None, l2}	None	None	None	None	None	None	None	None	None

**Table 5:** WCN-LSTM’s optimized hyper-parameter values.

Hyper-parameters	Description	Candidate Values		HIV4			LM			Vader		
				Time step			Time step			Time step		
				3	7	10	3	7	10	3	7	10
Dropout rate	Dropout rate for dropout layers	{0.2,0.35,0.5}		0.2	0.5	0.35	0.2	0.2	0.35	0.35	0.35	0.35
α	Weight for market news	{0.2,0.3, 0.4,0.5, 0.6}	**Where α + β + γ = 1**	0.4	0.5	0.5	0.4	0.4	0.6	0.5	0.5	0.4
β	Weight for sector news	{0.2,0.3, 0.4,0.5, 0.6}		0.4	0.2	0.2	0.2	0.2	0.2	0.2	0.2	0.2
γ	Weight for stock news	{0.2,0.3, 0.4,0.5, 0.6}		0.2	0.3	0.3	0.4	0.4	0.2	0.3	0.3	0.4

#### 5.3.3 Early stopping

In the training phase of the neural network, an epoch is considered a critical hyper-parameter. If the number of epochs is too high, then it can lead to overfitting of the training dataset. Whereas, a less number of epochs may get an underfit model.

Early stopping is a mechanism that controls the number of epochs by monitoring the performance measure and stops training when the model’s performance reaches the maximum. We have implemented this mechanism for baseline (LSTM) and WCN-LSTM prediction models and training stops when validation loss does not decrease in 10 consecutive epochs in order to get the regularized models.

After performing all the validation and optimization steps we have got optimized hyper-parameters values for all scenarios under consideration. By using these values, we have finalized models for all combinations of sentiment lexicon and time steps.

## 6. Experimental results and discussion

We have employed proposed and baseline models to perform experiments. Furthermore, we also conducted statistical testing for making quantitative decisions. Finally, the discussion is presented to answer research questions and to describe the importance of the proposed model.

### 6.1 Experimental results

Experiments are performed for three different time steps: 3, 7, and 10 previous days’ information, using three different sentiment dictionary scores. For the baseline model, input sequences are generated using the selected time step and concatenated using date, and then passed to the first LSTM layer in the baseline model.

For WCN-LSTM input sequences are generated using the same approach but they are not concatenated. All these sequences are aligned according to the stock transactions and news publishing dates. These sequences are input to the model using a unique path for each sequence.

Experimental results are shown in graphs. Where columns represent stocks from all leading sectors of PSX and rows represent the accuracy of prediction models for each sentiment dictionary.

#### 6.1.1 For time step = 3

**Fig 4:**
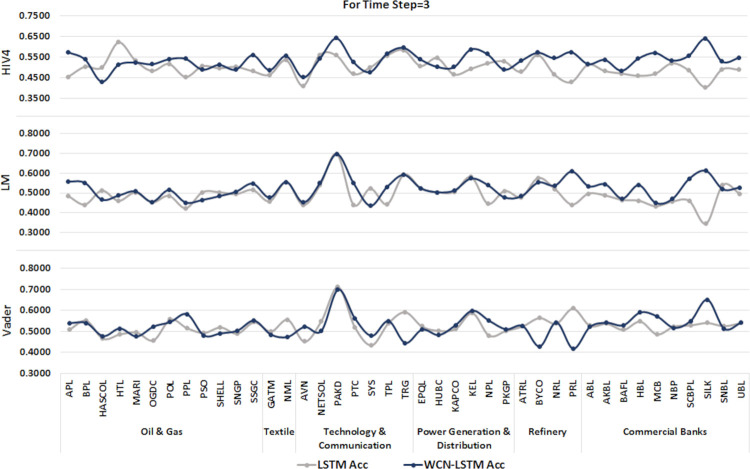
Accuracy of prediction models by incorporating information from the last 3 days using the test set.

**Fig 5:**
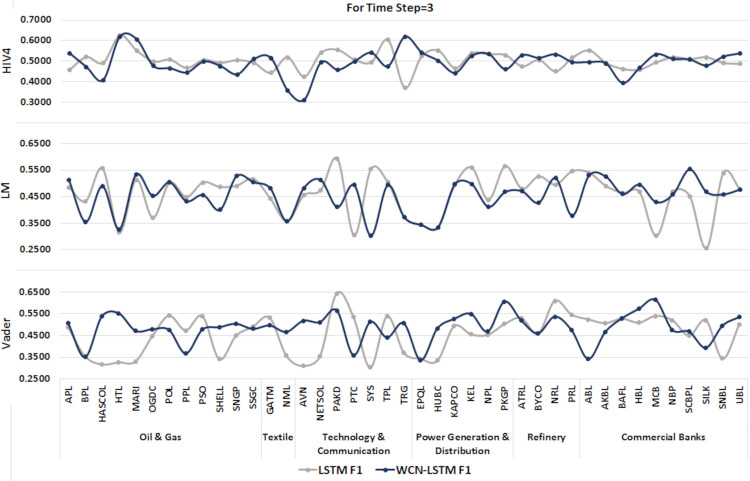
F1-score of prediction models by incorporating information from the last 3 days using the test set.

A time series sequence is generated using the stock data from the last three days of transactions along with stock-related news headlines. Experiments are performed using the proposed and baseline model using stock price and sentiment scores sequences calculated from three different sentiment dictionaries. It is shown in Figs 4 and 5.

It can be observed clearly that WCN-LSTM performs better in terms of accuracy for most of the stocks. Using the HIV4 sentiment dictionary, WCN-LSTM gets better accuracy for 32 out of 41, while using LM, 28 out of 41, and using Vader, 22 out of 41 stocks. For the F1-score, baseline performance is better than WCN-LSTM using HIV4 and LM sentiment dictionaries. The baseline model produces better F1-score for 26 stocks using HIV4 and 20 stocks using LM out of 41 stocks. While WCN-LSTM gives a better F1-score for 24 stocks using Vader. While some of the stocks from different sectors have the same accuracy and F1-score for both models.

The accuracy of WCN-LSTM for all three sentiment dictionaries can be analyzed in Fig 4. It is obvious that the accuracy of the proposed model using HIV4 is better than LM and Vader in most experiments. While sentiment scores calculated using the LM sentiment dictionary enhanced WCN-LSTM performance better than Vader lexicon.

6.1.2 For time step = 7

**Fig 6:**
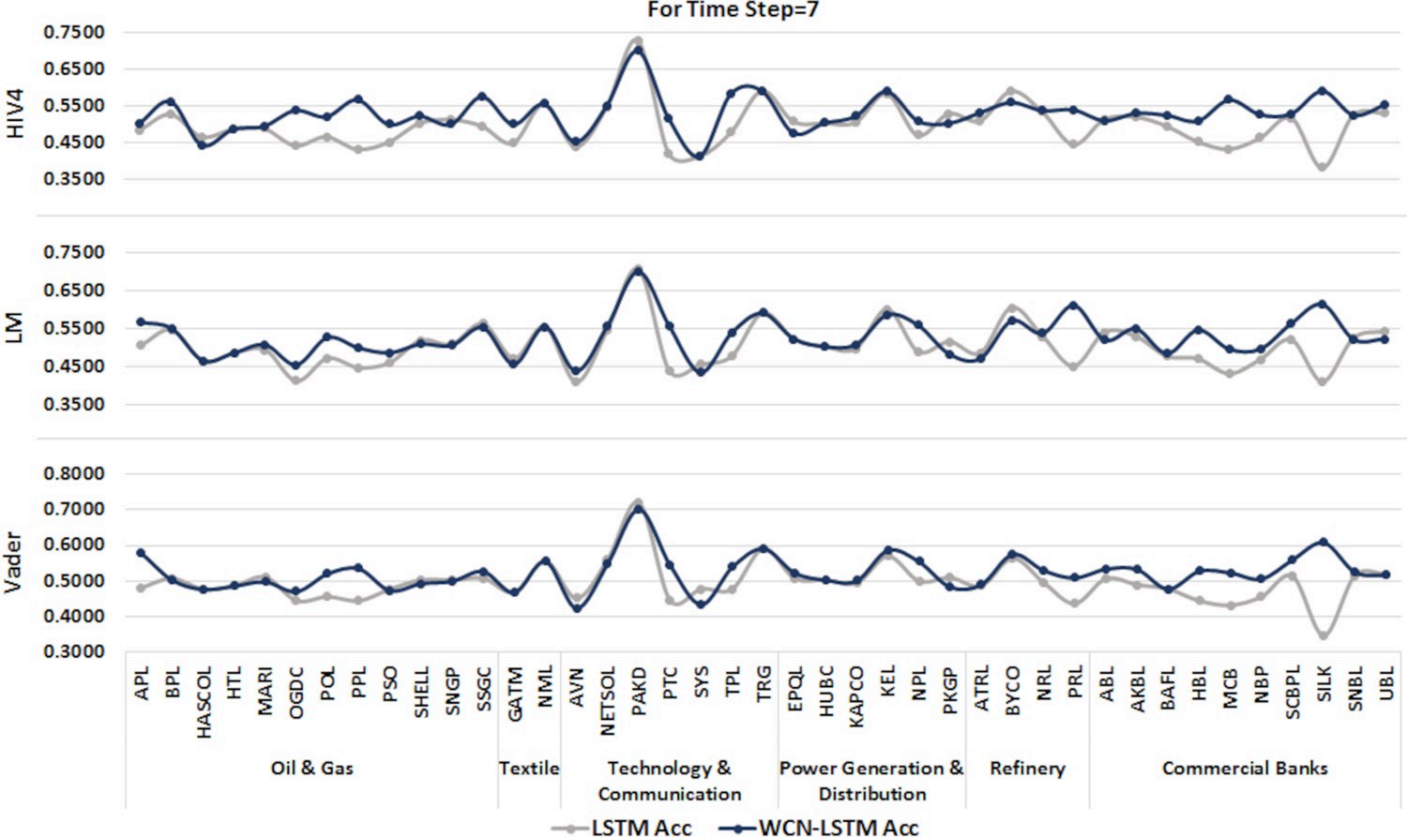
Accuracy of prediction models by incorporating information from the last 7 days using the test set.

**Fig 7:**
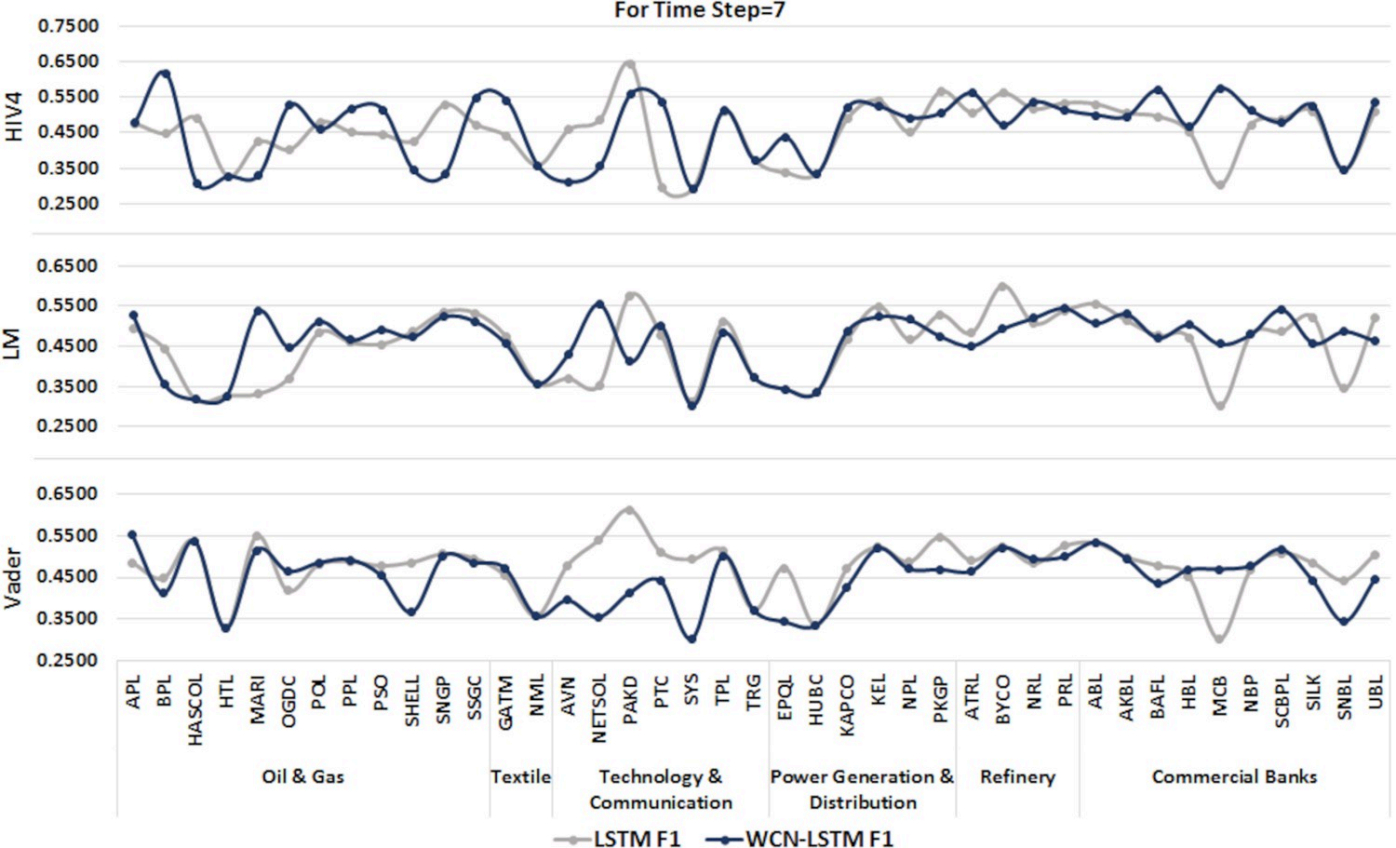
F1-score of prediction models by incorporating information from the last 7 days using the test set.

A time series sequence is generated using the data from the last seven days of transactions along with stock-related news headlines. Experiments are performed using a proposed and baseline model for sentiment scores calculated from three different sentiment dictionaries. Experimental results for time step 7 are illustrated in Figs 6 and 7.

It can be observed that WCN-LSTM performs better in terms of accuracy for most of the stocks. Using the HIV4 sentiment dictionary, WCN-LSTM gets better accuracy for 28 out of 41, while using LM and Vader, 23 out of 41 stocks. For the F1-score, WCN-LSTM produces better F1-score for 20 stocks using HIV4 and LM out of 41 stocks. While LSTM gives the better F1-score for 25 stocks using Vader. While some of the stocks from different sectors have the same accuracy and F1-score for both models.

The accuracy of WCN-LSTM for all three sentiment dictionaries is analyzed and it is observed that the accuracy of the model using HIV4 is better than LM and Vader in most experiments. While sentiment lexicons Vader and LM, equally influenced WCN-LSTM performance.

#### 6.1.3 For time step = 10

**Fig 8:**
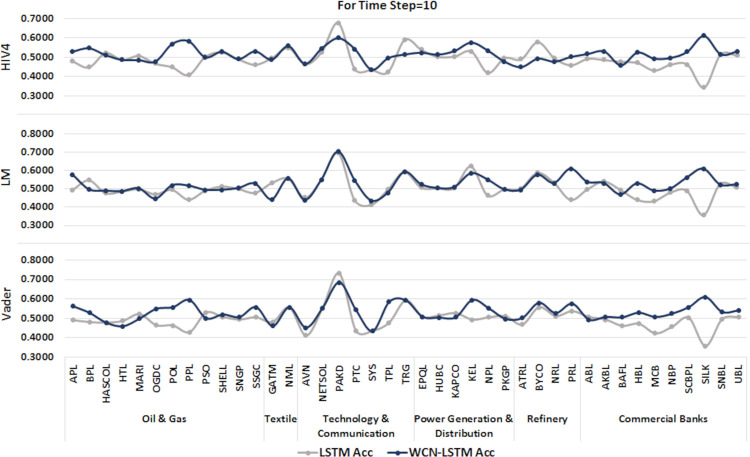
Accuracy of prediction models by incorporating information from the last 10 days using the test set.

**Fig 9:**
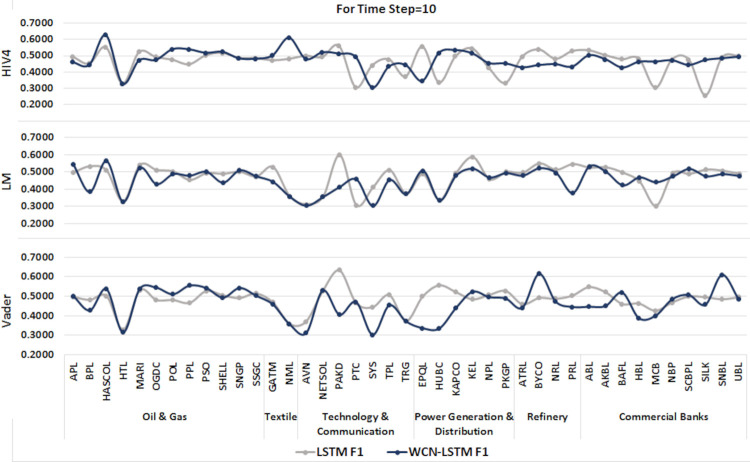
F1-score of prediction models by incorporating information from the last 10 days using the test set.

A time series sequence is generated using the data from the last 10 transactions days along with stock-related news headlines. Experiments are performed using proposed and baseline model for sentiment scores calculated from three different sentiment dictionaries. It is presented in Figs 8 and 9.

WCN-LSTM performs better in terms of accuracy for most of the stocks. Using HIV4 sentiment dictionary, WCN-LSTM gets better accuracy for 24 out of 41, while using LM, 22 out of 41, and using Vader, 26 out of 41 stocks. For the F1-score, baseline performance is better than WCN-LSTM using the HIV4, LM, and Vader sentiment dictionaries. The baseline model produces better F1-score for 24 stocks using HIV4 and 23 stocks using LM and Vader out of 41 stocks. While some of the stocks from different sectors have the same accuracy and F1-score for both models.

The accuracy of WCN-LSTM for all three sentiment dictionaries is analyzed using experimental results. WCN-LSTM performance using Vader is slightly better than HIV4. In the same way sentiment scores calculated using HIV4 produced better WCN-LSTM results than the LM sentiment dictionary.

### 6.2 Statistical analysis

We have adopted Wilcoxon signed-rank test to statistically compare whether the predictive performance of the two models is significantly different from each other. The Wilcoxon signed-rank test is a non-parametric and distribution free technique. It is considered safer than a parametric t-test due to the exemption in the assumption of normality and homogeneity of variance [39]. We have followed the work of [40–42] to use the Wilcoxon signed-rank test in order to comparatively analyze the predictive performance of WCN-LSTM and LSTM forecasting models.

We have constructed three hypotheses to statistically analyze the experimental results. For hypothesis testing, the model accuracy measure which is a continuous value is taken as a response variable. While for all three hypotheses, models, time steps, and sentiment lexicons are taken as independent variables. Our independent variable consists of two related groups where the same participants are presented in both groups. In order to conclude that one model performs better than the other, we have defined null and alternative hypotheses accordingly.

We have chosen 0.05 as a significant level or threshold value. To accept or reject the null hypothesis, the p-value is examined. If the p-value is less than a significant level, then the null hypothesis is rejected at a confidence level of 95%. The null hypothesis is accepted if the p-value is greater than the significance level. In the results, a p-value less than significance level is shown with ‘*’.

#### 6.2.1 First hypothesis

In our first hypothesis, we want to compare the prediction accuracy of WCN-LSTM and LSTM forecasting models denoted as Acc_WCN-LSTM_ and Acc_LSTM_ Our null hypothesis states that there is no significant difference between the predictive performance of both models. While the alternative hypothesis states that the predictive performance of WCN-LSTM is better than the LSTM model.

Null Hypothesis: H_0_: Acc_WCN-LSTM =_ Acc_LSTM_

Alternative Hypothesis: H_1_: Acc_WCN-LSTM >_ Acc_LSTM_

We have performed hypothesis testing for three different time steps and three different sentiment dictionaries and which is demonstrated in Table 6. According to Table 6, the predictive performance of WCN-LSTM was significantly better than the LSTM forecasting model in 7 out of 9 different scenarios.

**Table 6:** p-values for the first hypothesis test.

Time Step	Sentiment Lexicon		
	HIV4	LM	Vader
**t3**	0.000*	0.004*	0.591
**t7**	0.000*	0.010*	0.001*
**t10**	0.012*	0.064	0.000*

*Significant at 0.05 level

#### 6.2.2 Second hypothesis

In the second hypothesis, WCN-LSTM prediction accuracy is compared using different sentiment dictionaries for each time step. Because it doesn’t allow us to compare more than two groups so we have conducted multiple tests. The null hypothesis states that there is no significant difference appears in prediction accuracy by employing different sentiment lexicons in the WCN-LSTM model. The null and alternative hypotheses are given below:

Null Hypothesis (H_0_): Acc_WCN-LSTM (HIV4) =_ Acc_WCN-LSTM (LM),_

Acc_WCN-LSTM (HIV4) =_ Acc_WCN-LSTM (Vader),_

Acc_WCN-LSTM (LM) =_ Acc_WCN-LSTM (Vader)_

Alternative Hypothesis (H_1_): Acc_WCN-LSTM (HIV4) >_ Acc_WCN-LSTM (LM),_

Acc_WCN-LSTM (HIV4) >_ Acc_WCN-LSTM (Vader),_

Acc_WCN-LSTM (LM) >_ Acc_WCN-LSTM (Vader)_

According to Table 7, for time steps 3 and 7, sentiment lexicon HIV4 performs significantly better than LM and Vader sentiment lexicons.

**Table 7:** p-values for the second hypothesis test.

Time Step	Sentiment Lexicon		
	HIV4-LM	HIV4-Vader	LM-Vader
**t3**	0.004*	0.195	0.912
**t7**	0.000*	0.071	0.998
**t10**	0.777	0.987	0.948

*Significant at 0.05 level

6.2.3 Third hypothesis

In the third hypothesis, comparisons are conducted between the prediction accuracy of WCN-LSTM for the sequences formed using different time steps. The null hypothesis states that there is no significant difference appears in the prediction accuracy of WCN-LSTM by incorporating sequences formed using different time steps. The Wilcoxon signed-rank test performs comparisons in different combinations for more than 2 groups. Moreover, these tests are performed for each sentiment lexicon adopted in experiments. The null and alternative hypotheses for multiple combinations of comparisons are given below:

Null Hypothesis (H_0_): Acc_WCN-LSTM (t3) =_ Acc_WCN-LSTM (t7),_

Acc_WCN-LSTM (t3) =_ Acc_WCN-LSTM (t10),_

Acc_WCN-LSTM (t7) =_ Acc_WCN-LSTM (t10)_

Alternative Hypothesis (H_1_): Acc_WCN-LSTM (t3) >_ Acc_WCN-LSTM (t7),_

Acc_WCN-LSTM (t3) >_ Acc_WCN-LSTM (t10),_

Acc_WCN-LSTM (t7) >_ Acc_WCN-LSTM (t10)_

In order to perform Wilcoxon signed-rank test for third hypothesis, all test combinations for each sentiment lexicon are tested. It is shown in Table **8**, using the HIV4 sentiment lexicon, time steps 3 and 7 performs better than time step 10. While using the LM sentiment lexicon, time step 7 performs better than time steps 3 and 10. Moreover, using the Vader sentiment lexicon, there is no significant difference between all the three time steps.

**Table 8:** p-values for the third hypothesis test.

Sentiment Lexicon	Time Step		
	t3-t7	t3-t10	t7-t10
**HIV4**	0.232	0.001*	0.049*
**LM**	0.996	0.656	0.032*
**Vader**	0.089	0.470	0.974

*Significant at 0.05 level

### 6.3 Discussion

We have implemented WCN-LSTM and LSTM based prediction models in order to answer our empirical research questions. We have also compared WCN-LSTM, qualitatively with existing models to highlight its novelty. Our comparative findings from different perspectives are discussed below:

#### 6.3.1 The superiority of WCN-LSTM

By observing the experimental results illustrated in Figs 4–9, it has been observed that WCN-LSTM incorporates sentiment scores of categorized news along with weighted impact, achieving better accuracy than LSTM. In the statistical analysis phase, Wilcoxon signed-rank test is adopted in order to endorse the significance of weighed categorized news incorporated in the prediction model. In Table 6, it is shown that the alternative hypothesis is accepted. Therefore, the performance of WCN-LSTM is better than the LSTM prediction model.

**Fig 10:**
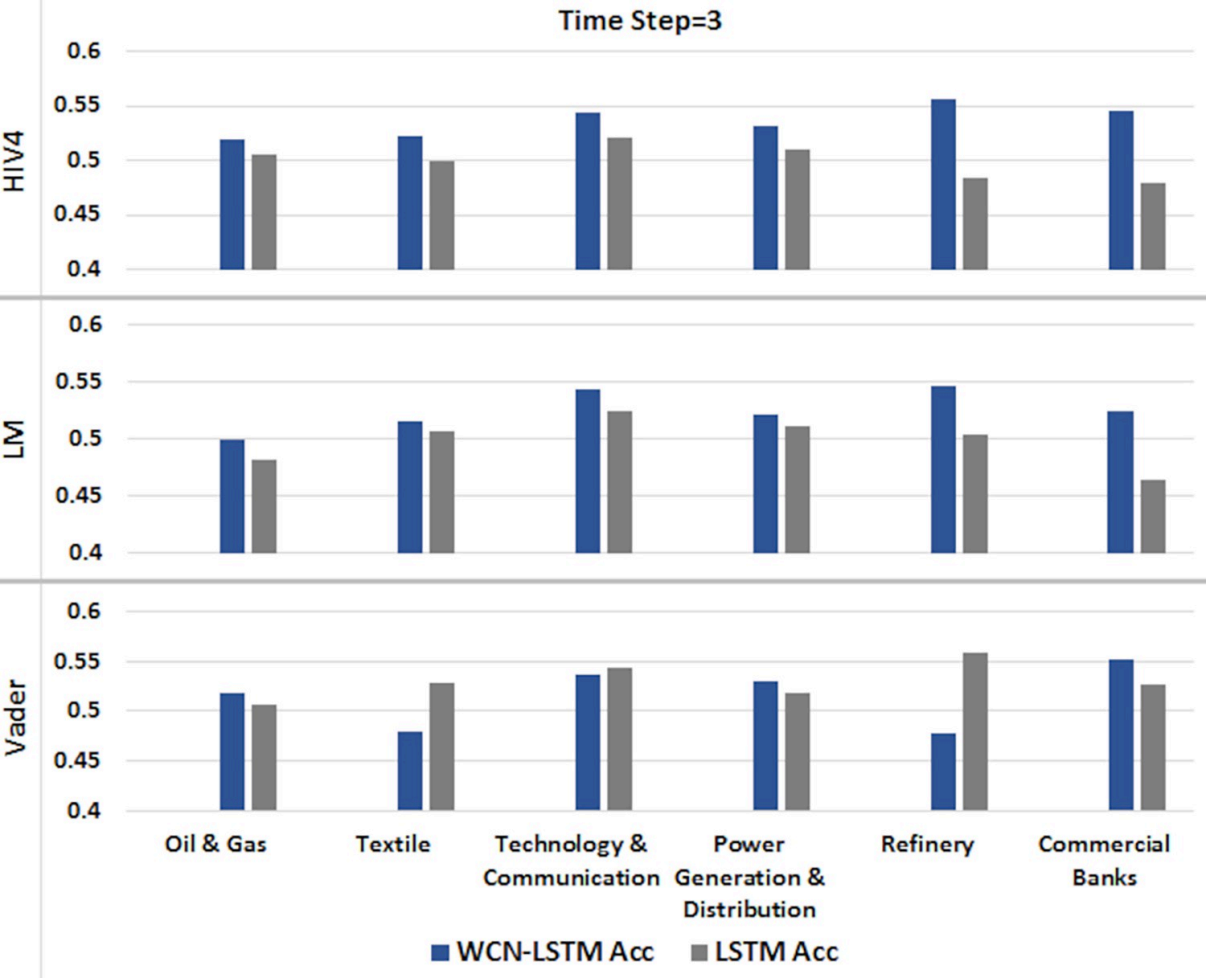
Comparison among sectors for time step = 3.

**Fig 11:**
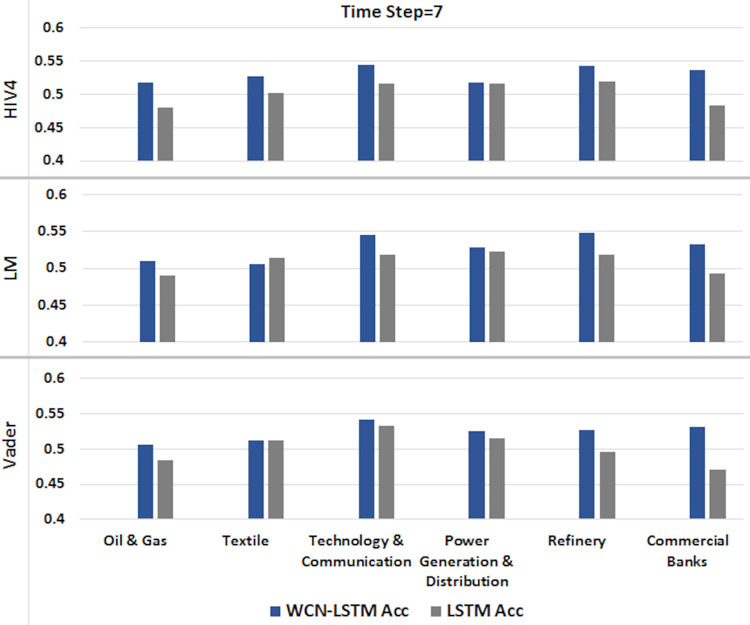
Comparison among sectors for time step = 7.

**Fig 12:**
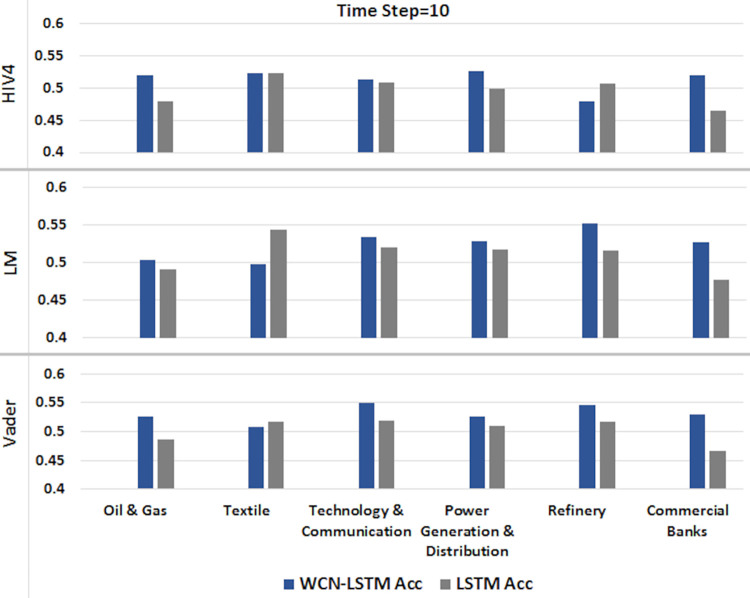
Comparison among sectors for time step = 10.

The performance of WCN-LSTM could also be observed at the sector level. The average accuracy achieved by WCNLSTM for each stock in a sector is calculated and compared from the baseline model. It could be observed in Figs 10–12, that WCN-LSTM clearly performs better than LSTM especially for sentiment lexicon HIV4 and time steps 3 and 7.

WCN-LSTM results could be analyzed for individual stocks to study the relationship between prediction accuracy and the number of news headlines in each news category. The number of market news is the same for all stocks while the number of sectors and stock-related news are different for each sector and stock. In Table 2, each news category is listed along with the number of news headline. In Figs 10–12, it is presented that Oil & Gas and Commercial Banks are two sectors with a large number of active stocks that scored better prediction accuracy produced by WCNLSTM in all cases of sentiment lexicon and time steps.

The sector Technology & Communication performs better except in one case. It could be deduced that the number of sector-related news headlines influenced positively on sector’s prediction accuracy. But this statement violated the case of the Textile sector where sector related new headlines are more than any other sector and the number of active stocks selected for experiments is less than any other sector. Furthermore, for the sector Commercial Banks number of stock related news are comparatively more than the any other sector’s stocks. In the case of PSX, it could be deduced that the large size of all news categories improves prediction accuracy at sector and stock level.

#### 6.3.2 Comparison between sentiment lexicons and input sequence length

The comparative analysis between sentiment lexicons is performed statistically and demonstrated in Table 7, in order to answer the research question RQ4. It reveals that HIV4 performed better than LM and Vader sentiment lexicons. Finally, experimental results disclosed that time step 3 and 7 for generating input sequences significantly enhanced WCN-LSTM performance. In Table 8, findings are statistically ascertained which answered the research question RQ5.

#### 6.3.3 Implications

**Fig 13:**
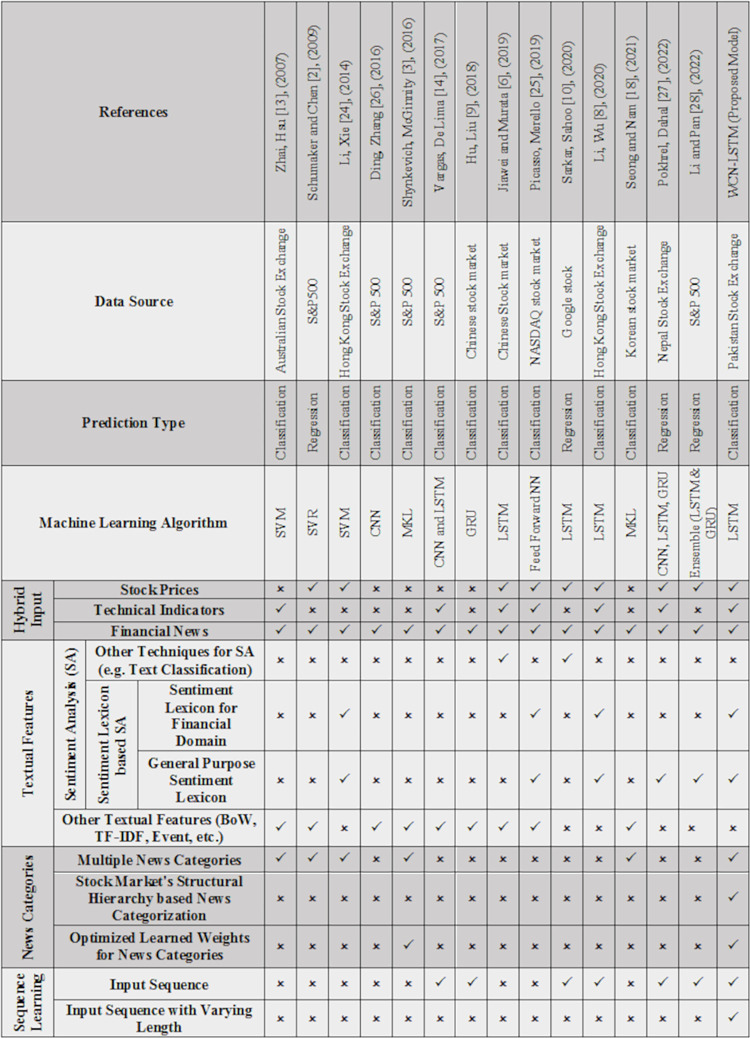
Comparison between WCN-LSTM and existing stock prediction model’s features.

In Fig 13, a qualitative comparison is presented in order to show the novelty of WCN-LSTM among the other state of the art stock prediction models. We have reviewed the existing work and identified the strength of hybrid input, sentiment analysis, and sequence learning in making predictions. Although the baseline approach incorporated all these features, but sequence length is not investigated in search of optimized length. Furthermore, we have found that there is a very rare attempt to group financial news according to their area of influence in the stock market and incorporate these news groups into the prediction model simultaneously. In existing work, the GICS standard is used to group financial news. However, it is revealed that GICS has a limitation in finding homogeneous news groups. By taking into account the existing opportunities and limitations, our proposed model WCN-LSTM employed hybrid input, lexicon-based sentiment analysis, and sequential learning. We have incorporated news groups related to the structural hierarchy of the stock market. We incorporated these news groups simultaneously into WCN-LSTM with learned weights. This is the first paper that suggests a sophisticated prediction model for the incorporation of new groups that are related to the structural hierarchy of the stock market. Furthermore, we have considered the case of PSX which is not explored yet by the research community for forecasting using hierarchical news groups. At a broader level, we have established a news sensitive stock prediction model that utilizes news groups that influence the market volatility with varying impact. For instance, news groups related to politics, terrorism, foreign affairs, natural disasters, etc., could also be incorporated into the prediction model according to their learned weights with minor adaptation.

## 7. Conclusion and future work

In this paper, we addressed the stock trend prediction problem by utilizing hybrid input, weighted news groups, sentiment analysis, and sequential learning. The LSTM layer is used to implement sequential learning, specifically designed to efficiently memorizes long and short-term dependencies in an input sequence. WCN-LSTM incorporates three news groups, namely market news, sector news, and stock news according to the structural hierarchy of the stock market. We have selected the case of the Pakistan Stock Exchange to conduct experiments. WCN-LSTM prediction model is adapted from LSTM based prediction model which we have considered as a baseline model to perform quantitative analysis. We have identified five empirical research questions. In order to answer these questions, experiments are performed using WCN-LSTM and baseline prediction models. Moreover, we also perform statistical analysis using Wilcoxon signed-rank test. We have shown that WCN-LSTM performed better than the baseline model. While for WCN-LSTM, sentiment lexicon HIV4 for time steps 3 and 7 performs satisfactorily among other candidate choices. To present the novelty of WCN-LSTM, we conduct a qualitative analysis by comparing our proposed model features with existing stock prediction models. However, there is a strong requirement for homogenous news groups. These news groups are required to learn their degree of influence on market dynamics before making predictions.

We have found that the LM sentiment lexicon did not perform well although specifically designed for the financial domain. To tackle this shortcoming, we are interested in adapting the sentiment lexicon for PSX using transfer learning based approaches like the Bert language model and incorporating it into our proposed WCN-LSTM model. We are also curious to examine other textual feature representations like word and event embedding along with knowledge bases that refines embedding. Other sources of textual features like company annual reports as well as other news groups related to politics, terrorism, and foreign policies could also be incorporated into the prediction model for further enhancement in prediction quality. Furthermore, there is much room to investigate hybrid architectures where the sequence learning model could be combined with other deep learning models to improve prediction results of our proposed model.

## Supporting information

S1 File (PDF)
