# Supplementary material for: LSTM based stock prediction using weighted and categorized financial news
Source: PLoS One. 2023 Mar 7;18(3):e0282234. doi: 10.1371/journal.pone.0282234 (PMC9990937; doi:10.1371/journal.pone.0282234)
Supplement: S1 File — (PDF) [file pone.0282234.s001.pdf]

News Headlines Categorization Scheme for Unlabelled Data

Shazia Usmani
Systems Research Laboratory,
FAST-National University of Computer and Emerging Sciences Karachi, Pakistan
shazia.usmani@nu.edu.pk

Jawwad A. Shamsi
Systems Research Laboratory,
FAST-National University of Computer and Emerging Sciences Karachi, Pakistan
jawwad.shamsi@nu.edu.pk

***Abstract*— Text categorization without training data is a difficult task and requires enough amount of hand labelled data to apply supervised methods, while manual labelling is a tedious job. In this paper a news categorization scheme is proposed to filter out and categorize news headlines related to Pakistan Stock Exchange (PSX) using negligible manual effort. By using domain knowledge, category names are selected manually then these category names are used as seed keyword to filter out news headlines. Natural Language Processing (NLP) based technique is used to extract context of seed keyword from initially filtered news headlines. These context terms are added in keyword list for string matching that further refines news filtration. Each news headline in a filtered news group is labelled and assigned a seed keyword term as a category label. Finally, a supervised classification technique is used to ensure the segregation of news categories as well as validates the performance of multiclass classification. Prepared dataset will be published in near future for potential uses explored by research community.**

Keywords— ***Text categorization, Unlabelled data, NLP, POS tagging***

I. INTRODUCTION

Proper categorization of news headlines is important in many aspects at our lives. Text categorization is a task of assigning a predefined category to a text on the basis of its contents [6]. A properly categorized news dataset could be used for analysis such as stock market prediction [2], [3], and [4]. News categorization is challenging in many aspects, first and foremost, it requires availability of relevant categorized dataset that can be used by the classifier to learn classification rules. Then extracted classification rules can be used by the classifier for the categorization of unlabelled data.

In stock trend prediction, news related to stock market, sectors, and stocks is definitely the relevant data to mine potential significant information about market volatility.

Pakistan Stock Exchange (PSX), is the official stock exchange of the country with trading centers working in big cities of Pakistan including Lahore, Karachi and Islamabad. General news archives are available to extract related news for Pakistan Stock Exchange but the big portion of these news corpus is not the relevant data for Pakistan stock market trend prediction. So, there is a need of properly categorized news headlines for overall PSX, sectors, and stocks. Furthermore, these news categories should be segregated enough so that their impact can be observed on the whole stock market, on individual sector and on individual stock separately.

In literature, a lot of work has been done related to the news categorization using training data. But there is no labelled data for PSX. In [1], a work has been done to capture news impact on stock market. News are grouped in eight categories using financial expert's manual efforts. These news categories are more general like financial, economic, foreign relations etc. These news are taken on weekly basis not on daily basis. Furthermore, this dataset is not public.

The contribution of this work is twofold. Firstly, a properly categorized news headlines dataset is created for PSX from the corpus of unlabelled news headlines. News categorization is performed using category name as a seed keyword. Then context related to these keywords are fetched using (Parts of Speech) POS tagging. On the basis of the final list of keywords, news headlines are filtered out accordingly. This categorized dataset is divided into training and test sets then a supervised classification method is used to ensure the segregation of each category. In Fig. 1, dataset preparation process is illustrated.

Secondly, this categorized dataset will be published for researchers. So that, it can be used from different aspect to explore PSX volatility using news headlines.

This paper is organized as follows: In Section II related work is discussed. Section III describes the complete process of data collection and preparation. Section IV validates finalized dataset. Finally, Section V concludes the paper.

II. RELATED WORK

The task of text categorization is dominated by the supervised techniques where large number of labelled training data is used. In [2], labelled data is used by a supervised approach to classify the news headlines into three categories. Then these categories are used to analyze the relation between news and stock price.

Copyright and Reprint Permission: Abstracting is permitted with credit to the source. Libraries are permitted to photocopy beyond the limit of U.S. copyright law for private use of patrons those articles in this volume that carry a code at the bottom of the first page, provided the per-copy fee indicated in the code is paid through Copyright Clearance Center, 222 Rosewood Drive, Danvers, MA 01923. For reprint or republication permission, email to IEEE Copyrights Manager at pubs-permissions@ieee.org. All rights reserved. Copyright ©2020 by IEEE.

Authorized licensed use limited to: UNIVERSITY OF BIRMINGHAM. Downloaded on May 10,2020 at 02:45:36 UTC from IEEE Xplore. Restrictions apply.

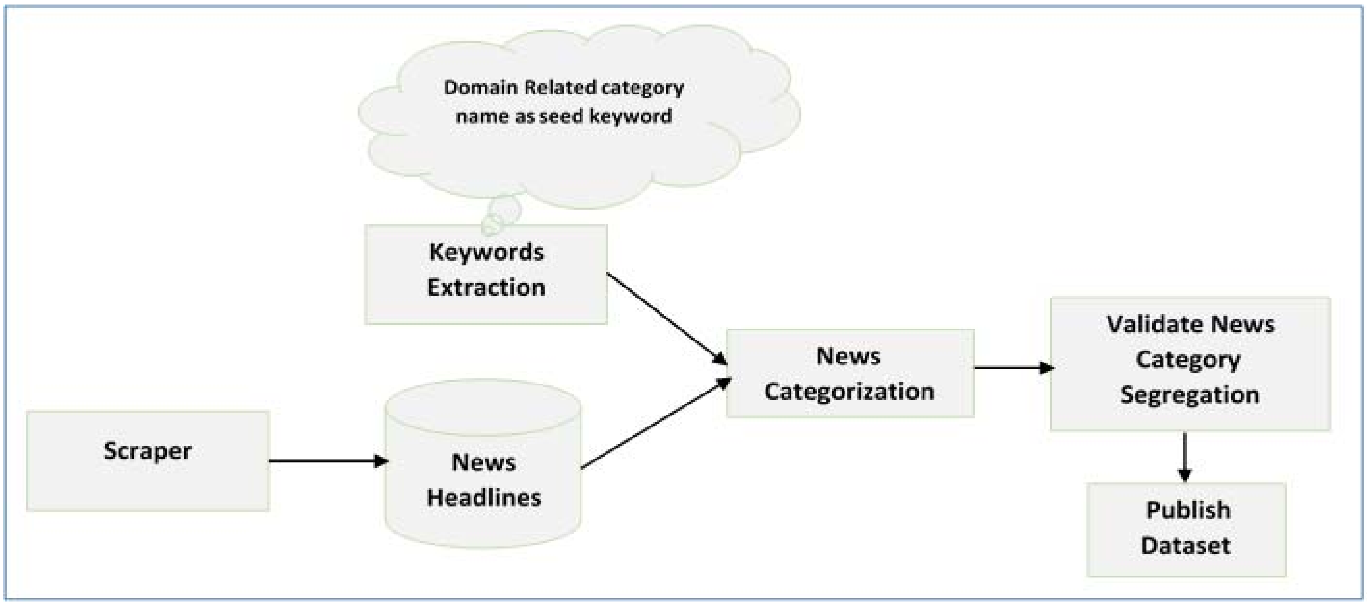

Fig. 1. Dataset Preparation Process

In [3], company specific news articles are considered for text categorization. These articles are labelled into four categories using an automatic process for text categorization using a hand-made thesaurus. In [13], [14] sentiment scores are used to categorize stock related news. This labelled news data is aligned with stock prices to forecast stock movements. In [15], news are labelled using sentiment scores then labelled data is validated by two economic scientists.

Labelled data can be prepared using manual filtration on the basis of some domain based constraints. In [1], news are categorized into different category using manual effort by domain experts.

In order to reduce manual effort for text categorization, many approaches have been proposed in literature. These approaches initially rely on manually provided category related keyword list. Then these keywords are used for text categorization based on similarity measure between documents and keywords per category. Then manual effort is reduced by providing category name as initial keyword. In [7], further improvement for reducing manual effort is achieved by providing category name as only input keyword for text categorization. Then Latent Semantic Analysis (LSA) based similarity and Word-Net based similarity are multiplied to calculate the final similarity score of document with category name. Results showed improvement in precision with Reuters-10 corpus. With further improvements and adaptation in lexical references and context model presented in [7], a category name as an initial input based categorization scheme is presented in [8].

III. Data Collection And Preparation

The News International, is the largest English language newspaper in Pakistan. Firstly, we selected publically available "The News" archives for daily news headlines[1]. News archived dataset is available from 2006 to date.

A. *Data Collection*

In literature, it is discussed that news headlines are more useful for stock prediction than complete news articles [5]. Scraper is developed to scrape news headlines from 2006 to 2018 and to store it in CSV file format. These news headlines are grouped in different categories like Business, Top Stories, Sports, World, Karachi, Islamabad, Lahore, and Peshawar. Each row of news headlines dataset is aligned with its publishing date. Table I illustrates some rows of scrapped data.

Table I presents example of news headlines from every category. It is observed that Top Story, World, and Business are those special categories which have news related to financial market. So finally, Top Story, World, and Business categories are selected to consider for data preparation.

B. *Data Preparation*

The selected news corpus contains around 2.5 million news headlines. News corpus word cloud is illustrated in Fig. 2. Word Cloud is a technique to show which words are the most frequent among the given text [9].

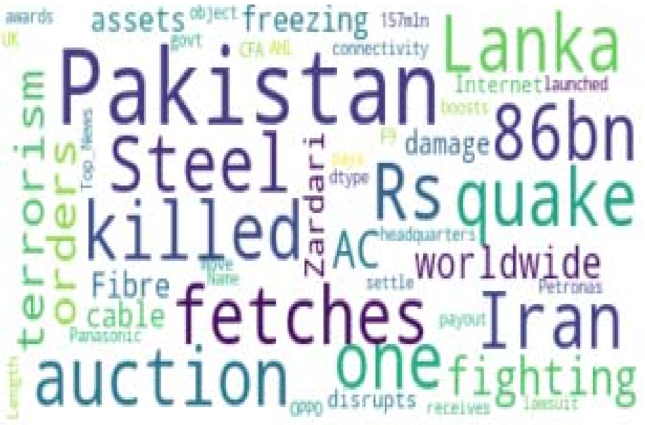

Fig. 2. Word Cloud for News Corpus

[1] https://www.thenews.com.pk/todaypaper-archive/

Authorized licensed use limited to: UNIVERSITY OF BIRMINGHAM. Downloaded on May 10,2020 at 02:45:36 UTC from IEEE Xplore. Restrictions apply.

TABLE I. THE NEWS HEADLINES

Date	Category	Headlines
01/04/2006	Top Story	Pakistan Steel fetches Rs 21.86bn at auction
01/04/2006	Top Story	70 killed in Iran quake
01/04/2006	World	Local polls boost Lankan president in peace bid
01/04/2006	World	EU gives Serbia month to nab war crimes fugitives
01/04/2006	National	SU to launch CCTV, FM radio soon
01/04/2006	National	China not a threat in region: Mushahid
01/04/2006	Sports	Delhi Blues beat PCB Greens women
01/04/2006	Sports	Dravid hails Raina for match-winning knock
01/04/2006	Business	Shamshad says exports will reach $28bn in 2010
01/04/2006	Business	CCOP approves LOA for second highest bidder for PAFL
01/04/2006	Karachi	Early completion of uplift projects stressed
01/04/2006	Karachi	Muttahida, PML agree on working formula
01/04/2006	Islamabad	Returning Afghan DPs asked to avail land distribution programme
01/04/2006	Islamabad	Nawaz preparing party for next polls
01/04/2006	Lahore	Hashmi pays tributes to Gilani
01/04/2006	Lahore	Abu Dhabi Tower scam Two confess to depriving people of millions of rupees
01/04/2006	Peshawar	ATC releases 23 alleged rioters on bail
01/04/2006	Peshawar	PHC enhances imprisonment of convict

The word cloud in Fig. 2 doesn't show the highest frequency words that are generally related to stock market.
This shows that stock market related keywords are required to filter out relevant news headlines using string matching. For keyword based text categorization, manual generation of keyword lists for each category is required. Initially, category name is taken as a seed keyword to filter out news for relevant category [9, 10]. Then this initial keyword is used to extract further related keywords for specific category [8].

- Assigning Category Name as a Seed Keyword

  In first step, category name is taken as a seed keyword for string matching [9, 10]. The names of categories are taken from the official website of PSX[2]. The name of stock market, its sector names, and stock names are taken as category names for news headlines categorization. In Table II, some categories name and their description are mentioned.

- Extracting Context of Seed Keywords

  Categorization by using the seed keywords alone may increase false positive rate. So there is a need to enrich seed keywords by further inspection of dataset under consideration. It is achieved by extraction of further category related keywords in the context of seed keyword. And then the final list of keywords is used to filter out news headlines for relevant category. Context related keywords are extracted by creating a POS tree for each sentence.

[2] https://www.psx.com.pk/

TABLE II. SOME CATEGORIES NAME & DESCRIPTION FROM DATASET

Category Name	Description
PSX or KSE	Due to historical reasons, PSX mostly known as KSE
Oil and Gas	Oil and gas sector
Textile	Textile Sector
Mari	Mari is stock in oil and gas sector
PSO	PSO is stock in oil and gas sector

The POS tagging is a standard Natural Language Processing (NLP) technique. All NLP implementations have this feature but spaCy, which is an NLP open source library is chosen. For instance, the top 10 nouns and verbs co-occur with the seed keyword "kse" are shown in Figure 3.

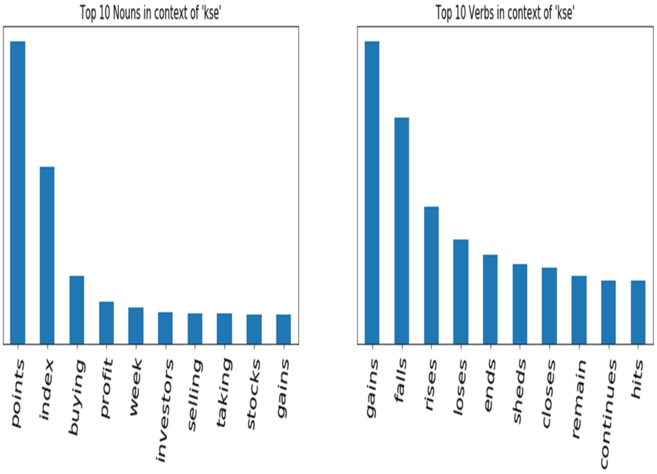

Fig. 3. Top 10 Nouns, and Verbs in the Context of Seed Keyword "kse"

- Finalizing List of Keywords for News Filtration

  Now, list of keywords, that is a seed keyword, verb and nouns in the context of seed keyword are used for further processing. Different combination of these keywords are used to filter out news headlines using Python string matching.
  Moreover, if news are filtered for specific sector of PSX, then all stock symbols are also added in the list of keywords for that category. Since news related to the sector's stock is also relevant news headline for that sector. Stock symbols are taken from official website of PSX. For instance, list of keywords for oil and gas sector is "oil and gas", "oil prices", "mari", "ogdc", "ppl", "pol", etc. . For the category of stock related news, only stock symbol is used as a keyword, for example, news for stock "PSO" is filtered using the keyword {"pso"}. In Fig. 4, some of the news headlines are shown using the keyword "oil price" for the category "Oil and Gas Sector".

- News Filtration and Categorization

  News filtered using a keyword's list for specific category are combined into a group while duplicate news are discarded. Publishing date of every news is

Authorized licensed use limited to: UNIVERSITY OF BIRMINGHAM. Downloaded on May 10,2020 at 02:45:36 UTC from IEEE Xplore. Restrictions apply.

also fetched. Then, a category label is assigned to all news in a group. For instance, word cloud for different categories are illustrated in Fig. 5, 6 and 7.

‘Oil prices fall',
'Oil prices up by 2.5 to 7.17pc',
'Hike in oil prices criticised',
'Oil prices rise above $73 per barrel',
'High oil prices to stay, says US energy secretary',
'Shell sees profits climb on soaring oil prices',
'Oil prices recede on easing Iran tension',
'Oman repaying foreign debt due to high oil prices',
'Oil prices march higher',
'Oil prices rebound $1 after sell-off',
'Oil prices rebound'

Fig. 4. News headlines form "oil and Gas" Category

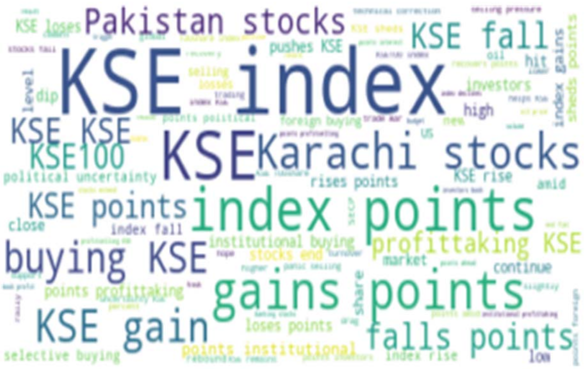

Fig. 5. Word Cloud for Category "KSE"

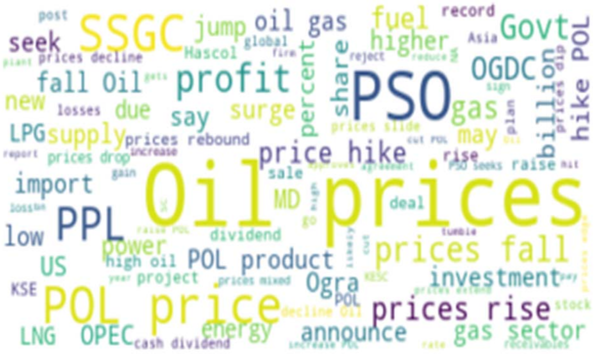

Fig. 6. Word Cloud for Category "Oil and Gas"

- Dataset Finalization and Potential Uses

Initially, the uncategorized dataset contains 2.5 million news headlines. For this work, PSX, some leading sectors, and stocks are considered as a category. After performing all the news categorization steps discussed above, around 11k news headlines for different categories of PSX are labelled and fetched. Remaining news headlines are irrelevant for PSX and not included in final dataset.

In Fig. 8 all steps of keyword based news headlines categorization scheme are shown.

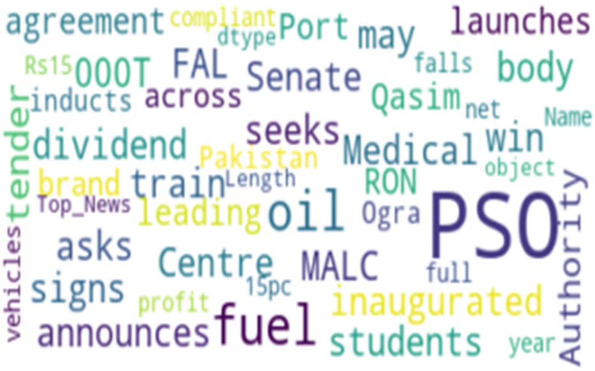

Fig. 7. Word Cloud for Category "PSO"

This dataset contains properly categorized news headlines for PSX with publishing date. Researchers can use this dataset to capture news headlines impact on stock market volatility from different aspects. News headlines are segregated in three groups. The first group contains news headlines for whole PSX, the second group combines news headlines for specific sector, and the third group has news headlines for specific stock. News impact can be observed from whole stock market to specific sector and specific stock separately. Moreover, these news impacts can be combined to observe collective impact of all news groups in market volatility. The dataset will be published in near future.

Input: Set of category names as a seed keyword
Input: Unlabelled news headlines corpus
Output: Categorized news headlines dataset

Step 1: Initialize a set of keyword list using category name for each category.
Step 2: Extend keyword list using nouns and verbs used in the context of seed keyword.
Step 3: Perform string matching to filter out all news headlines where a keyword from a keywords list occurred.
Step 4: Combine the search result for the whole keyword list and discard duplicate news headlines. Assign category name as a label to each news headline.
Step 5: Extract all labelled news headlines as a final dataset and ignore all unlabelled news headlines.

Fig. 8. Category Name Based Categorization Scheme

IV. Validation of News Categories Segregation

Text classification is one of the fundamental task in NLP. With recent advancement in neural networks, deep neural networks are considered more promising for text classification than shallow models [12].

In order to validate news categories segregation, a fully connected artificial neural network based classification model is employed.

Authorized licensed use limited to: UNIVERSITY OF BIRMINGHAM. Downloaded on May 10,2020 at 02:45:36 UTC from IEEE Xplore. Restrictions apply.

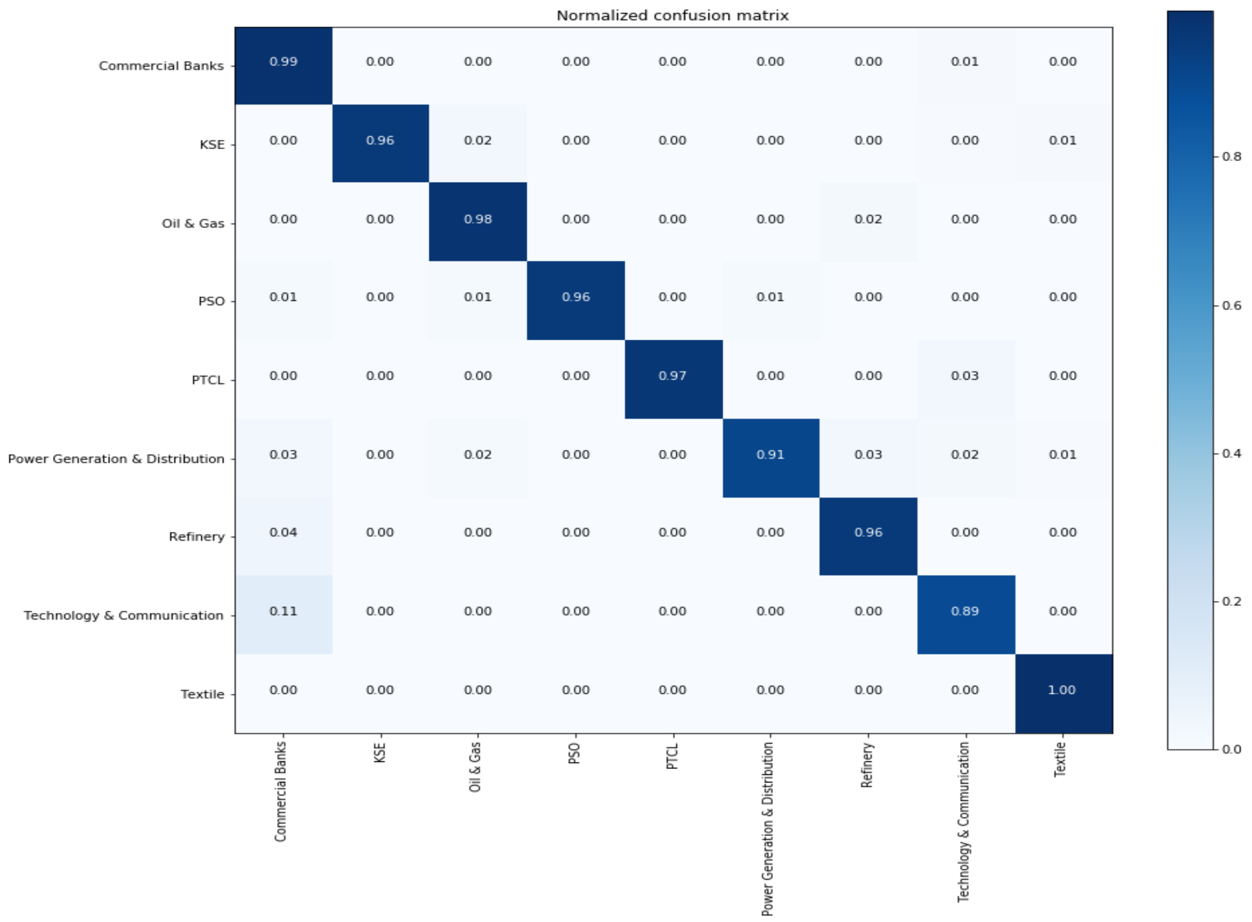

Fig. 9. Normalized Confusion Matrix for News Headlines Categorization

The classification model contains 2 hidden layers with softmax activation function in output layer. For multiclass classification problem, softmax activation function is used specifically in output layer.

Softmax outputs a vector that represents the probability distributions of a list of classes that is used to find out the class which has maximum probability. This classification model is implemented using Keras library written in Python. Keras, is an open source library that provides user friendly environment to enable fast experimentation with deep neural networks [10].

Model's parameters are tuned in a traditional way using simple trial and error method for parameter tuning.

For experiment, dataset is divided into training and test set. Model validation is performed using Keras feature for automatic validation [10]. To evaluate model performance, normalized confusion matrix is used. With imbalanced classes in dataset, normalized confusion matrix is a good technique to summarize the performance of classification technique. It visually interprets results, where a row represents an actual class and a column represents predicted class [11].

Initially, experiment is performed for nine news headlines categories. In Fig. 9, it is observed that all classes have accuracy more than 88 percent although the data is imbalanced. This shows the degree to which categories are segregated in dataset. A properly categorized dataset enhances classification model performance and it is clearly shown in Fig. 9.

V. Conclusion

In this paper, news headlines categorization scheme is presented while there is no training data. The scheme can be applied to any categorization problem in which categories are described by initial sets of seed keywords and provided dataset is unlabelled. The presented scheme extracts the domain related category name as a seed keyword with negligible manual effort. The proposed scheme utilizes NLP based techniques to extract context of seed keyword that is used to further refine the categorization scheme's results.

Furthermore, final dataset is validated using ANN based supervised multiclass classification technique and demonstrated using normalized confusion matrix.

Acknowledgment

This research work is supported by Higher Education Commission (HEC), Islamabad, Pakistan.

References

[1] S. Raza, and K. M. Ali, "Daily Stock Market Movements: From the Lens of News and Events," No. id: 12188, Feb 2017.

Authorized licensed use limited to: UNIVERSITY OF BIRMINGHAM. Downloaded on May 10,2020 at 02:45:36 UTC from IEEE Xplore. Restrictions apply.

[2] S. Takahashi, T. Masakazu, T. Hiroshi, and T. Kazuhiko, "Analysis of the relation between stock price returns and headline news using text categorization," International Conference on Knowledge-Based and Intelligent Information and Engineering Systems. Springer, Berlin, Heidelberg, Sep 2007.

[3] M. Mittermayer and F. K. Gerhard, "Newscats: A news categorization and trading system," Sixth International Conference on Data Mining (ICDM'06), IEEE, Dec 2006.

[4] Z. Hu , L. Weiqing, B. Jiang, L. Xuanzhe, and L. Tie-Yan, "Listening to chaotic whispers: A deep learning framework for news-oriented stock trend prediction," Proceedings of the eleventh ACM international conference on web search and data mining, Feb 2018.

[5] M. R. Vargas, D.L. Beatriz SLP, and G. E. Alexandre, "Deep learning for stock market prediction from financial news articles," International Conference on Computational Intelligence and Virtual Environments for Measurement Systems and Applications (CIVEMSA). IEEE, Jun 2017.

[6] R. Jindal, M. Ruchika, and J. Abha, "Techniques for text classification: Literature review and current trends," webology , vol. 12.2, Dec 2015.

[7] L. Barak, D. Ido and S. Eyal, "Text categorization from category name via lexical reference," Proceedings of Human Language Technologies: The 2009 Annual Conference of the North American Chapter of the Association for Computational Linguistics, Companion Volume: Short Papers, Jun 2009.

[8] C. Liebeskind, K. Lili and D. Ido, "Text categorization from category name in an industry-motivated scenario," Language resources and evaluation, vol. 49.2, pp. 227-261, Jun 2015.

[9] R Soni, S Sharma, H Fagna and S Mittal, "News Analysis Using Word Cloud," Advances in Signal Processing and Communication. Springer, Singapore, pp. 55-64, 2019.

[10] J. Moolayil, Moolayil, and J. Suresh, “Learn Keras for Deep Neural Networks,” Apress, 2019.

[11] Parkhi, Omkar M., Andrea Vedaldi, Andrew Zisserman, and C. V. Jawahar, "Cats and dogs." Conference on computer vision and pattern recognition. IEEE, Jun 2012.

[12] C Du, Z Chen, F Feng, L Zhu, T Gan and Nie, L, "Explicit interaction model towards text classification," Proceedings of the AAAI Conference on Artificial Intelligence., vol. 33. Jul 2019.

[13] S. Mohan, Saloni, S. Mullapudi, S. Sammeta, P. Vijayvergia, and D. C. Anastasiu, "Stock Price Prediction Using News Sentiment Analysis," Fifth International Conference on Big Data Computing Service and Applications (BigDataService), IEEE, Apr 2019.

[14] R. Batra, and D. S. Muhammad, "Integrating StockTwits with sentiment analysis for better prediction of stock price movement," International Conference on Computing, Mathematics and Engineering Technologies (iCoMET), IEEE, Mar 2018.

[15] S.F. Yazdani, A.A.M. Masrah Azrifah, M.S.Nurfadhlina, P.S. Yashwant and R.A.L.Ahmed, "Sentiment classification of financial news using statistical features," International Journal of Pattern Recognition and Artificial Intelligence , vol. 31.03, 2017.

Authorized licensed use limited to: UNIVERSITY OF BIRMINGHAM. Downloaded on May 10,2020 at 02:45:36 UTC from IEEE Xplore. Restrictions apply.
